# Polyphenol-Based Nanomedicine: Versatile Platforms for Immune Modulation and Therapeutic Delivery

**DOI:** 10.3390/molecules31061051

**Published:** 2026-03-22

**Authors:** Quoc-Viet Le, Trinh K. T. Nguyen, Ngoc-Nhi Phuong, Dai-Phuc Phan Tran, Van-An Duong, Hien V. Nguyen, Phuoc-Quyen Le, Huy Truong Nguyen, Minh-Quan Le

**Affiliations:** 1 Research Group in Pharmaceutical and Biomedical Sciences, Faculty of Pharmacy, Ton Duc Thang University, Ho Chi Minh City 72912, Vietnam; lequocviet@tdtu.edu.vn (Q.-V.L.); nguyentruonghuy@tdtu.edu.vn (H.T.N.); 2 School of Pharmacy, University of Medicine and Pharmacy at Ho Chi Minh City, Ho Chi Minh City 70000, Vietnam; ntktrinh.ths.cndpbct24@ump.edu.vn; 3 Faculty of Pharmacy, Ton Duc Thang University, Ho Chi Minh City 72912, Vietnam; phuongthingocnhi@tdtu.edu.vn (N.-N.P.); tranphandaiphuc@tdtu.edu.vn (D.-P.P.T.); 4 The Brown Foundation Institute of Molecular Medicine, McGovern Medical School, The University of Texas Health Science Center at Houston, Houston, TX 77030, USA; van.an.duong@uth.tmc.edu; 5 Faculty of Pharmacy, Van Lang University, Ho Chi Minh City 70000, Vietnam; hien.nv@vlu.edu.vn; 6 Department of Pharmacy, Ajou University, Suwon 16499, Republic of Korea; phuocquyenle@ajou.ac.kr

**Keywords:** polyphenol, nanoparticle, immunotherapy, inflammation, cancer, metal–polyphenol

## Abstract

Polyphenols, abundant compounds found in natural sources, exhibit various biological activities, including immunomodulatory properties that can either stimulate or suppress immune responses, making them promising for therapeutic applications. However, their poor solubility, low bioavailability, rapid metabolism, and non-specific distribution require advanced drug delivery strategies to overcome limitations in clinical translations. Therefore, nano-drug delivery systems have been intensively studied to explore the full therapeutic potential of polyphenols. Distinct from conventional paradigms where polyphenols serve solely as active compounds, this review advances the concept of polyphenol-based nanomedicine as dual-functional platforms: bioactive structural components and intrinsic immune modulators. Recent strategies to improve the loading efficacy of polyphenols, enhance their cellular uptake, prolong circulation, and enhance specific delivery based on those nanocarriers are emphasized. In addition, polyphenol-based nanoparticles, in which polyphenols serve as structural components, were also studied as self-therapeutics or multifunctional nanocarriers for drug delivery. We intensively focus on their immunomodulatory applications and highlight their potential in preclinical as well as clinical settings for the treatment of various diseases and therapeutic purposes, including autoimmune diseases, cancer immunotherapy, vaccination, inflammation, and infectious diseases. Although polyphenol nanoparticle development has made significant advances, there remain challenges in formulation stability, unclear in vivo toxicity profiles, and clinical translation. Further studies on optimizing nanoparticle design and assessing long-term toxicity are necessary to materialize their application. A combination of polyphenol nanoparticles with other immunotherapies may promise a pronounced efficacy and safety profile.

## 1. Introduction

Our immune system is a powerful defense barrier, playing important roles in both protection and pathogen elimination. A strong immune response can help eliminate pathogens and cancer cells, while dysregulation may result in autoimmune diseases or tumor evasion. Unlike conventional treatments that target the diseased cells and tissues, immunotherapies aim to boost host defense and restore immune balance. Recently, various immunotherapies have gained considerable attention due to their high specificity, long-lasting effects, and reduced systemic toxicity compared to chemotherapy. However, they still face challenges in terms of efficacy and safety profile in certain populations, including but not limited to the resistance to immune checkpoint inhibitors, cytokine release syndrome of chimeric antigen receptor T-cell therapy, poor stability, suboptimal endosomal escape of mRNA vaccines, or dose-limited toxicity of cytokine therapies.

**Figure 1:**
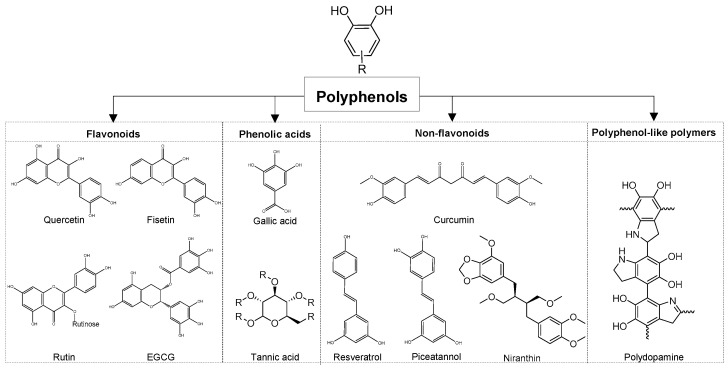
Representative polyphenols categorized by structure.

Polyphenols are a group of abundant compounds found in fruits, vegetables, and medicinal plants. Natural polyphenols are categorized in four main groups based on chemical structure, including flavonoids, phenolic acids, stilbenes, and lignans (Figure 1). In addition, polyphenols can also exist as oligomeric or polymeric structures, giving rise to polyphenol-based polymers derived from monomeric polyphenols [1]. The general structure of polyphenols consists of aromatic rings with one or more hydroxyl groups. They are recognized as a powerful source of anti-oxidant, anti-inflammatory, and immunomodulatory activities. Although numerous studies have examined their immunomodulatory effects and potential applications in disease treatment, polyphenols still face significant challenges in clinical translation due to poor bioavailability, short half-life, and limited targeting ability. Nano-drug delivery systems have emerged as novel tools to overcome the therapeutic limitations of polyphenols. In addition, these compounds, per se, play pivotal roles as multifunctional blocks in the construction of potential polyphenol-based carriers that improve therapeutics, including chemical and biological drugs.

Indeed, polyphenols can interact with drug molecules through charge interactions, hydrogen bonding, and hydrophobic interactions, such as π–π stacking and van der Waals interactions. They can self-assemble into nanosized complexes by acting as crosslinkers when interacting with other macromolecules such as proteins, nucleic acids, and polysaccharides. Polyphenols can strongly interact with proteins via hydrogen bonding, owing to the presence of multiple phenolic groups in their structures, which act as both proton donors and acceptors at neutral pH. Polyphenols also form complexes with nucleic acids through hydrophobic interactions and hydrogen bonding, which can intercalate between nucleic acid bases. In addition to those binding mechanisms, cationic polysaccharides can be condensed with polyphenols via charge interaction with phenolate groups. Various small drug molecules can interact with polyphenols via multiple binding modes, such as hydrogen bonding and π–π stacking, thereby preventing crystallization and improving aqueous solubility. Multivalent metal ions can also chelate with polyphenols and induce self-assembly into nanocomplexes. These interaction mechanisms feature polyphenols as a competitive carrier candidate for drug delivery. Of note, most of the driving force from polyphenol-drug interactions is non-covalent and can be dissociated in the presence of other competitive factors in the environment. Therefore, drug–polyphenol complexes could be biodegradable and suitable for in vivo applications [2].

This review focuses on immunomodulatory effects of polyphenols, particularly highlighting recent strategies for developing functional polyphenol nanoparticles as both therapeutics and drug delivery systems. The review will cover applications in immune regulation, specifically in the context of cancer immunotherapy, autoimmune diseases, vaccines, inflammation, and immune disorder-related diseases. Perspectives on the current challenge in bench-to-bedside translation and the future development of these emerging polyphenol nanoparticles will be discussed.

## 2. Polyphenols and Immunomodulatory Activities

Polyphenols can modulate immunity toward either enhancing immune response or suppressing excessive immune activation. These immunomodulatory effects are mediated via complex interactions with various immune cell types. The mechanism of immunomodulation is broadly influenced by key signaling pathways, cytokine secretion, regulation of gene expression, and immune cell interaction (Table S1).

### 2.1. Polyphenols Regulate Dendritic Cells’ Activities

Dendritic cells (DCs) are critical antigen-presenting cells that serve as a bridge between the innate and adaptive immune responses. Activation of immunogenic DCs contributes to developing anti-cancer immunity by triggering antigen-specific T cell responses, while the excessive or dysregulated activation of DCs may lead to pathological inflammation, breakdown of self-tolerance, immune exhaustion, and therapeutic failure. Hence, modulation of DCs activity represents a crucial strategy in cancer immunotherapy, and the treatment of inflammatory diseases [3].

Polyphenols exhibit immunoregulatory effects on DCs by tuning intracellular signaling networks that govern DC functional states, protecting DCs from oxidative stress-induced dysfunction while reshaping their immune phenotypes. In cancer therapies, polyphenols can enhance DC-mediated immunogenicity indirectly by inducing immunogenic cell death (ICD) to activate anti-tumor immunity. Epigallocatechin gallate (EGCG) promoted DC maturation and enhanced the effector function of CD8+ T cells in colorectal cancer. For instance, EGCG-induced immunogenic cell death promoted the exposure and release of damage-associated molecular patterns, leading to increased phagocytosis and activation of DCs, thereby facilitating CD8^+^ T-cell activation and reshaping the tumor immune microenvironment [4].

Conversely, in autoimmune and inflammatory conditions, multiple polyphenols promote tolerogenic DC phenotypes. Silibinin and curcumin suppressed the activation of DCs, prevented them from antigen presentation, while enhancing their capacity for endocytosis. Besides that, silibinin also inhibited the production of pro-inflammatory cytokines such as Interleukin (IL)-12, IL-23, and tumor necrosis factor-alpha (TNF-α) induced by lipopolysaccharide (LPS) [5,6].

Overall, these findings demonstrate that polyphenols can modulate DC function in both immunogenic and immunotolerant states. This immunoregulation supports the development of polyphenol-based nanosystems that directly target DCs, either to enhance antigen presentation and immune activation in cancer immunotherapy and vaccination, or to induce immune tolerance in autoimmune and inflammatory diseases.

### 2.2. Polyphenols Modulate Macrophage Activation and Phenotype Differentiation

Macrophages are crucial cells of the innate immune system, playing essential roles in host defense, tissue repair, and immune regulation. Depending on microenvironmental cues, macrophages can undergo functional polarization toward an M1 macrophage (pro-inflammatory) or an M2 macrophage (anti-inflammatory). During the inflammatory phase, M1 macrophages release TNF-α, (IL-1β, IL-6, and various inflammatory cytokines to eliminate pathogens and amplify inflammatory responses. However, the accumulation of M1 macrophages during a prolonged inflammatory phase delays healing due to excessive inflammatory responses. In contrast, in the proliferative phase, M2 macrophages secrete large amounts of IL-10, transforming growth factor-β (TGF-β), and other anti-inflammatory cytokines to suppress the inflammatory cascade, contribute to trigger tissue regeneration, recovery of tissue integrity, and functions [7].

One of the major challenges in immunomodulatory therapy is controlling the production of cytotoxic agents, such as reactive oxygen species (ROS). ROS released from M1 macrophages is essential for antimicrobial activity; however, excessive production can exacerbate inflammatory signaling and cellular damage. Polyphenols have emerged as effective modulators of macrophage-mediated inflammation due to directly removing ROS and modulating the activity of enzymes responsible for removing ROS [8]. Curcumin, gallic acid, and resveratrol could promote polarization of macrophages toward an anti-inflammatory M2 phenotype, including IL-10 production, CD206 expression, and mitochondrial oxidative metabolism, while dramatically inhibiting the release of pro-inflammatory mediators (TNF-α, IL-6, and IL-1β). These effects are mediated through interconnected pathways such as the phosphorylation of phosphoinositide 3-kinase (PI3K) and protein kinase B (AKT) (PI3K–Akt), activation of endogenous protective pathways TLR4/MyD88/NF-κB, and SIRT1/Nrf2 signaling, highlighting promoting M2 macrophage polarization, thereby facilitating tissue repair and functional recovery [9,10,11,12,13,14]. Importantly, the immunomodulatory effects of polyphenols are highly context dependent. In the tumor microenvironment, polyphenols can reprogram tumor-associated macrophages toward immune-activating phenotypes. Tannic acid could regulate the reprogramming of tumor-associated macrophages from the M2 phenotype to an anti-tumoral, immune-activating phenotype, suppress tumor cell proliferation, and new blood vessel formation [15].

Overall, polyphenols exhibit a flexible capacity to modulate macrophage polarization and inflammatory signaling in a microenvironment-dependent manner. These properties are useful for preparing polyphenol-based nanomedicines that directly target macrophages to control inflammation and promote tissue repair in immune-related disorders.

### 2.3. Polyphenols Regulate T Cell Activation and Differentiation

T cells are the central mediators of both humoral and cellular adaptive immune responses, tightly regulated to ensure effective host defense while maintaining immune tolerance. Dysregulated T-cell responses can result from multiple mechanisms, leading to the pathogenesis of autoimmune diseases, chronic inflammatory disorders, and tumor immune evasion. Among T cell subsets, Treg cells play a critical role in the formation of immune tolerance. By secreting anti-inflammatory cytokines, including IL-10, TGF-β, andIL-35, Treg cells restrain autoreactive T cells and prevent immune-mediated tissue damage. Thus, Treg-based modulation represents a promising therapeutic strategy for autoimmune disorders [16].

Polyphenols have been shown to ameliorate various autoimmune diseases by enhancing Treg cells activation or inhibiting inflammatory factors in chronic inflammation and tumor-associated immune dysregulation. For example, curcumin could delay the development of multiple sclerosis through stimulating Treg and T helper type 2 (Th2) cell polarization, accompanied by increasing the expression of IL-10 production, transcription factors, and gene expression that are able to regulate over-immune reactions [17,18,19]. The increase in IL-10 also promoted the polarization of macrophages towards the anti-inflammatory M2 phenotype. Thereby attenuating pathological immune activation in autoimmune and inflammatory disease models.

Beyond Treg induction, polyphenols also suppress pathogenic Th17-driven inflammation. Procyanidin B2 gallate, procyanidin B2 3,3″-di-O-gallate (PCB2DG), quercetin, and fisetin controlled the cytokine network associated with Th17 cells and interacted directly with the glutamine transporter to inhibit glutamine influx, thus reducing intracellular glutamine levels in CD4+ T cells and decreasing the number of interferon-gamma (IFN-γ) cytokines, thus exerting potential therapeutic effects in inflammatory diseases or autoimmune disorders [20,21,22,23]. Oleuropein increased the frequency of CD4^+^CD25^+^FoxP3^+^ regulatory T cells (CD4^+^CD25^+^FoxP3^+^ Tregs) and induced the production of IL-10 and TGF-β [24].

In contrast, within the tumor immune microenvironment, regulatory immune cells often contribute to immune evasion. In the tumor immune microenvironment, Treg cells secrete immunosuppressive cytokines, including TGF-β and IL-10, which impair anti-tumor activity. Therefore, it is necessary to selectively suppress immunoregulatory cells to restore antitumor immunity.

Polyphenols, promising immunomodulatory agents, exhibit immunomodulatory and antitumor effects by various mechanisms, including reprogramming the tumor immune microenvironment, restoring T cell-mediated antitumor immunity, directly inhibiting tumor cell proliferation, inducing apoptosis, and suppressing tumor angiogenesis. Resveratrol, which exhibits immunomodulatory effects, enhances anti-tumor activity by reducing CD8^+^CD122^+^ Tregs and M2 macrophages, inhibiting tumor cell proliferation, and inducing apoptosis of tumor cells [25,26]. Besides that, resveratrol inhibited Arginase-1 and C-X-C chemokine receptor type 2 (CXCR-2) expression in murine MDSCs and, thus, reversed T cell-mediated immune response, reduced the immunosuppressive properties of TCDD-induced MDSCs [27].

To sum up, given their potential to modulate T cell activation and differentiation, polyphenols can be used to develop polyphenol-based nanomedicines to restore immune tolerance or enhance antitumor T cell immunity.

### 2.4. Polyphenols Suppress Neutrophils to Reduce Inflammation

Neutrophils are innate immune cells that are recruited to sites of infection to host defense against bacterial disease, due to the release of effector molecules such as reactive oxygen species, myeloperoxidase, and neutrophil extracellular traps [28]. In addition to their antimicrobial functions, neutrophils play a critical role in tissue repair and the resolution of inflammation by engulfing and degrading extracellular fragments and allowing for optimal organ repair [29]. However, excessive neutrophil activation has been associated with increased host pathology through collateral organ damage. Therefore, it is crucial to regulate neutrophil activity effectively to maintain a balance between antimicrobial defense and inflammatory resolution in developing a therapeutic platform.

Polyphenols have been shown to regulate neutrophil-mediated inflammation through their antioxidant and signaling-modulatory properties. By strongly inhibiting ROS production and modulating redox-sensitive intracellular pathways, polyphenols can attenuate excessive neutrophil activation while preserving their essential host defense functions. Similarly, gallic acid has demonstrated protective effects in chronic obstructive pulmonary disease (COPD) by alleviating neutrophil-driven inflammation. Gallic acid was suggested to reduce symptoms of COPD exacerbations by inverse modulation of redox-sensitive transcription factors, specifically nuclear factor-κB (NF-κB) and nuclear factor erythroid 2-related factor 2 (Nrf2), which decreased neutrophil numbers. This dual regulation of inflammatory and antioxidant pathways highlights the capacity of polyphenols to rebalance dysregulated neutrophil responses in chronic inflammatory diseases [30].

Resveratrol has also been reported to attenuate neutrophil-mediated oxidative stress and inflammatory responses by inhibiting endothelin-mediated autocrine signaling. Mechanistically, resveratrol was demonstrated to reduce oxidative stress and the inflammatory response of neutrophils via inhibition of endothelin autocrine signaling by suppressing the extracellular signal-regulated kinase signaling pathway [31]. These effects further support the role of polyphenols in modulating neutrophil signaling cascades implicated in inflammation-associated tissue injury, supporting their potential use in polyphenol-based nanotherapeutic strategies aimed at controlling excessive inflammation while preserving host defense.

### 2.5. Polyphenols Enhance NK Cells’ Activities

Natural killer (NK) cells are key components of the innate immune system, playing a crucial role in early defense against viral infections and malignant transformation through contact-dependent cytotoxic activity and cytokine secretion, interferon-gamma (IFN-γ). However, the activation of NK cells should be precisely regulated, as dysregulated or uncontrolled activation can lead to immune exhaustion, tissue damage, and aberrant inflammatory responses, thereby compromising antiviral and antitumor immunity [32]. Conventional immunostimulatory agents often induce transient or non-specific activation, underscoring the need for modulators that can fine-tune NK cell responses in a context-dependent manner.

Polyphenols have emerged as promising immunomodulators that directly stimulate NK cells and cytokine production while preserving immune homeostasis, helping them kill virus-infected or cancerous cells more effectively. Among them, resveratrol has been extensively investigated for regulating both innate and adaptive immune responses, including NK cell activation. Resveratrol enhanced NK cell-mediated antiviral and antitumor responses through modulating intracellular signaling pathways associated with immune cell metabolism, survival, and effector function. Through modulating the mammalian target of rapamycin (mTOR) pathway and increasing cellular Myb (c-Myb) expression, resveratrol effectively inhibited tumor growth and metastasis. Resveratrol activated NK cells and innate immune lymphocytes by enhancing the secretion of IFN-γ, leading to increased NK cell cytotoxicity in conjunction with IL-2 [33]. In parallel, resveratrol also demonstrated antiviral immunomodulatory activity by regulating NK cell-mediated antiviral responses, contributing to the suppression of viral replication and enhanced immune defense. These findings highlight the ability of resveratrol to potentiate innate immune mechanisms critical for early viral clearance [34].

These studies indicated that polyphenols could modulate NK cell function across both antiviral and antitumor contexts by enhancing cytotoxicity and cytokine production through metabolic and transcriptional regulation. The incorporation of polyphenols into nanomedicine, as drugs or drug carriers, allows sustained and localized modulation of NK cell activity, enhancing therapeutic efficacy while minimizing systemic immune-related adverse effects in cancer and viral diseases.

## 3. Enhanced Delivery of Polyphenols by Nanoparticles

**Figure 2:**
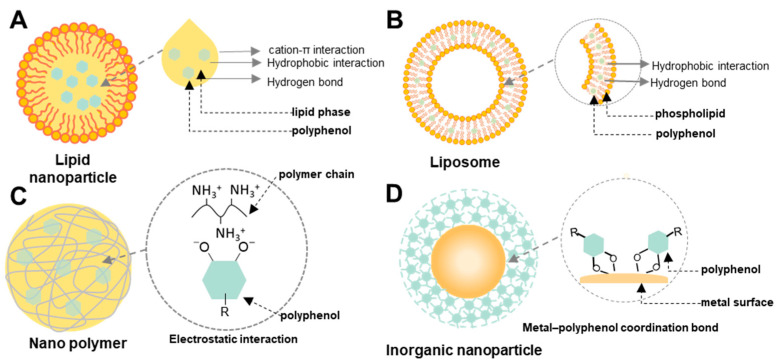
Nanoparticles for polyphenol delivery. In lipid nanoparticles (**A**) and liposomes (**B**), polyphenols are physically entrapped in the hydrophobic domains, thus enhancing their stability, solubility, and protection against degradation. In polymeric nanoparticles (**C**), polyphenols are incorporated via hydrophobic interactions, hydrogen bonding, and π–π stacking with the polymeric matrices, whereas in inorganic nanoparticles (**D**) (e.g., metal oxides), they can enable surface adsorption or covalent bonding, forming stabilized polyphenol shells.

Although polyphenols have shown numerous potential benefits in disease treatment, they still face challenges in clinical translation as therapeutics due to poor absorption, broad distribution, and certain toxicities. Therefore, various drug delivery systems have been developed to improve the treatment outcome of polyphenols (Figure 2 and Table 1).

### 3.1. Lipid-Based Nanoparticles

Lipid-based nanoparticles (LNPs) are versatile drug delivery systems constructed from lipid molecules organized into nanoscale structures (Figure 2A) [59,60]. They are widely used for the delivery of drugs, genes, and vaccines, with notable success in COVID-19 mRNA vaccines [61,62]. LNPs provide several advantages, including protection of therapeutic agents from in vivo degradation, enhanced solubility and efficacy, regulated drug release, and precise delivery to target sites. These biocompatible carriers can encapsulate both hydrophilic and hydrophobic substances. LNPs can be categorized into liposomes, nanoemulsions, and solid lipid nanoparticles (SLNs) [63]. Different lipid-based platforms should be selected based on the physicochemical characteristics and therapeutic goals of specific polyphenols.

Liposomes are spherical, nanoscale vesicles consisting of one or more phospholipid bilayers surrounding an aqueous core. They are used as drug delivery systems with high biocompatibility, high drug encapsulation, and the ability to control and target drug release [64]. Hydrophilic drugs are loaded into the aqueous core, while hydrophobic compounds are embedded within the lipid bilayers (Figure 2B), helping to protect therapeutic agents from degradation [65]. Moreover, their surface can be modified with various ligands or polymers to further enhance their stability and target specificity [66]. Some polyphenols, such as curcumin or resveratrol, have demonstrated potential in preventing and treating inflammation. However, the therapeutic use of curcumin or resveratrol is limited by their poor stability and low water solubility, as well as their low bioavailability in the gastrointestinal environment. Therefore, loading curcumin or resveratrol into liposomes is an ideal solution for local administration or oral treatment. These liposomes were developed by the thin-film hydration method, and curcumin or resveratrol was encapsulated between the lipid bilayers. These nanoparticles exhibited particle sizes of 100–200 nm, demonstrating high cellular uptake, potential for prolonged circulation, and tumor tissue enrichment. Liposome co-loading curcumin and (5-Fluorouracil) 5-FU exhibited good biocompatibility, prolonged circulation, and gastrointestinal accumulation in zebrafish. They showed superior tumor cytotoxicity in HT-29, HCT-116, and HGC-27 cell lines compared to single-drug formulations or physical mixtures [35]. The resveratrol-liposomes showed the capacity to re-educate the inflammatory macrophages from M1- to M2-like phenotype, scavenged ROS, and inhibited the NF-κB signaling and inflammasome activation, reducing the pro-inflammatory cytokines IL-1β, IL-6, and TNF-α in a ligature-induced periodontitis mouse model [36].

Besides loading into liposomes, using nanoemulsion to deliver polyphenols is another suitable strategy, thanks to the high kinetic stability and tunable droplet sizes; therefore, it can improve tissue penetration for cosmeceutical applications [67,68,69,70]. In a study by Ao et al., resveratrol (RES) and fish oil were encapsulated in nanoemulsions, utilizing hyaluronic acid-poly(glyceryl)10-stearate as an emulsifier to improve stability [38]. The resveratrol-nanoemulsion had a particle size of 215 nm and a high Encapsulation Efficiency (EE) (96.5–98.2%), enhanced stability for both fish oil and RES, and greatly augmented oxidative stability and free radical scavenging capacity across diverse environmental conditions. Moreover, to enhance the stability of the formulation, nanoemulsions loaded with RES can be further developed into nanogels. A resveratrol-loaded nanoemulgel (RES-NEG) was developed by spontaneous nano-emulsification for managing atopic dermatitis in a mouse model [37]. The RES-NEG was prepared with a SEPINEO™ P 600 gel base and propylene glycol, which exhibited higher skin retention of RES in an ex vivo study as compared with the free RES-loaded gel. In 2,4-DNCB-induced atopic dermatitis mice, the topical application of RES-NEG significantly improved the atopic dermatitis skin condition. In the skin tissues, the real-time polymerase chain reaction (PCR) analysis revealed a significant reduction in pro-inflammatory cytokines. Thus, the RES-NEG enhanced RES solubility and retention in the skin, thereby enhancing RES efficacy against atopic dermatitis.

In addition, SLNs are nanocarriers produced from biocompatible and biodegradable lipids in a solid state at room and body temperature [71,72]. The solid matrix enhances the stability of SLNs, encapsulates both hydrophilic and hydrophobic drugs, making them effective alternatives to other colloidal drug delivery systems like liposomes, nanoemulsions, and polymeric NPs [73,74,75]. SLNs could load rutin, resveratrol by using the solvent emulsification/diffusion method [39,40]. Rutin or resveratrol was efficiently encapsulated within SLNs, obtaining high encapsulation efficiency and drug content. Rutin-SLNs showed dose-dependent antioxidant activity in human glioblastoma astrocytoma (U373) culture cells, especially at higher doses (50 μM), compared to the free drug. In SKBR3/PR cells, the D-α-Tocopheryl polyethylene glycol 1000 succinate-resveratrol-solid lipid nanoparticles (TPGS-Res-SLNs) demonstrated higher cytotoxicity compared to free RES and RES-SLNs, likely due to enhanced solubility and cellular uptake. These SLNs inhibited cell migration, induced mitochondrial dysfunction, and enhanced tumor treatment efficacy by promoting apoptosis. In SKBR3/PR xenograft tumor models, TPGS-RES-SLNs induced greater tumor apoptosis and improved therapeutic outcomes than free RES.

Overall, lipid-based nanocarriers should not be viewed as generic delivery systems, but as formulation tools strategically matched to the physicochemical limitations and immunomodulatory functions of polyphenols. Such rational design enables enhanced stability, bioavailability, and immune-targeted efficacy, although further in vivo studies are required to fully assess long-term safety and clinical translational potential.

### 3.2. Polymeric Nanoparticle

Polymer-based nanoparticles have emerged as versatile and effective platforms in drug delivery, offering enhanced solubility, stability, controlled release, and targeted delivery. Their excellent stability, uniform particle size, and high drug-loading capacity make them particularly suitable for polyphenol encapsulation (Figure 2C) [76]. Nanoparticles used for the delivery of polyphenols can be classified into natural and synthetic polymer-based systems, each with distinct properties that influence their biocompatibility, biodegradability, and drug release [77].

Among natural polymers used to prepare nanoparticles for polyphenol delivery, chitosan is widely studied for its mucosal adhesion, biocompatibility, biodegradability, and ability to enhance the solubility and stability of polyphenolic compounds [78,79]. The cationic nature of chitosan facilitates electrostatic interactions with negatively charged polyphenols, while hydrogen bonding between hydroxyl groups of polyphenols and amino/hydroxyl groups of chitosan contributes to high encapsulation efficiency and sustained release [80,81]. Thus, polyphenol (rutin, resveratrol, curcumin, epigallocatechin gallate (EGCG), and urolithin B) loaded chitosan nanoparticles (NPs) were prepared in order to prepare a controlled and targeted delivery system [43,44,45,82,83,84]. Rutin-encapsulated chitosan nanoparticles by the ionic gelation method showed the particle size (approximately 92 nm) and the interaction of the amino group of chitosan with a negatively charged site at mucin enhanced the permeation of rutin and exhibited excellent mucoadhesive strength. In vivo evaluations in a cerebral ischemia rat model revealed improved neurobehavioral activity, reduced infarction volume, and enhanced brain-targeting efficiency (1443.48%), demonstrating the superiority of rutin-loaded chitosan nanoparticles over free rutin [45]. Similarly, resveratrol-loaded chitosan nanoparticles exhibited a controlled release profile in vitro and demonstrated enhanced cytotoxicity against MCF7 and SKBr3 breast cancer cell lines as well as greater antioxidant activity than free resveratrol [43]. To further optimize performance, chitosan can be combined with other polymers like Pluronic F127, PVA, or beta-cyclodextrin. These hybrid nanoparticles showed improved encapsulation efficiency for curcumin or Urolithin B and strongly demonstrated antibacterial or anticancer activity in vitro [41,44]. In addition to chitosan, other natural polymers, such as sodium alginate, gelatin, and zein (a corn-derived protein), have also been explored to improve the hydrophobic nature of certain polyphenols [46,85,86]. Rutin loaded into zein nanoparticles exhibited high entrapment efficiency (~88%) along with prolonged and controlled drug release. These nanoparticles exhibited superior antioxidant activity due to a synergistic effect between zein and rutin and showed enhanced cellular uptake [46].

While natural polymers are particularly suited for improving the bioavailability of polyphenols through biocompatible interactions, synthetic polymers offer greater control over release kinetics and targeting precision. Poly(lactic-co-glycolic acid) (PLGA) is a widely used synthetic polymer with its biocompatibility, biodegradability [87]. PLGA nanoparticles enhanced the stability and therapeutic efficacy of polyphenols such as rutin, led to significantly reduced hepatic nodules, enhanced antioxidant enzyme levels, and improved biochemical and histopathological parameters in hepatocellular carcinoma models [48]. In addition to PLGA, Eudragit S100 (polymethyl methacrylate) has also been used to prepare rutin-loaded nanoparticles to improve rutin colon-targeted delivery of rutin and prolonged release compared to free rutin [49]. Notably, polymeric nanoparticles can also exploit the intrinsic bioactivity of polyphenols beyond their role as drug carriers. Tannic acid could serve as a modifying ligand, directing the specific accumulation of delivered drugs. Tannic-acid-modified PLGA loaded with tacrolimus exhibited accumulation in lymph nodes and significantly increased the localized concentration of tacrolimus in a heart transplantation model, decreased rejection grades, and a marked extension of graft survival time [47].

To enhance the stability and dispersibility of the nanoparticles, Lin and colleagues prepared curcumin-loaded zein nanoparticles and coated them with carboxymethylated short-chain amylose, which helped prevent aggregation and improve gastric resistance for targeted delivery to the intestine [42]. Mostafa et al. developed chitosan-coated PLGA nanoparticles for the encapsulation of cranberry powder extract [88]. The chitosan coating enhanced encapsulation efficiency, mucoadhesion, and intestinal permeability while promoting cellular uptake. These improvements are due to chitosan’s positive charge, which helps overcome PLGA’s negative surface charge and facilitates interaction with negatively charged cell membranes.

### 3.3. Inorganic Nanoparticles

Inorganic NPs are a class of nanocarriers made from non-carbon-based materials, such as metals, metal oxides, and silica [89]. These NPs have gained significant attention in biomedical, environmental, and industrial applications due to their unique properties, including high surface area, tunable size, and stability [90]. They also possess enhanced mechanical strength, thermal stability, and conductivity compared to organic NPs. In the context of drug delivery, inorganic NPs can encapsulate various therapeutic agents and provide controlled release, reducing side effects and improving treatment efficacy (Figure 2D) [91].

Gold-based nanostructures are among the most extensively studied inorganic platforms for polyphenol integration. EGCG-capped gold nanoparticles (EGCG@AuNPs), which were exposed to a polyphenol and Zn(II) solution to form a mesoporous metal–phenolic network (MPN) at pH ~8 (MPN@AuNPs) [50]. With a particle size of 45.4 nm and a large mesoporous surface area, these particles enabled penetration across an in vitro blood–brain barrier model and efficient sequestration of amyloid-β peptides, significant potency against amyloid aggregation and toxicity in vitro. Similarly, resveratrol-loaded 198gold nanoparticles (RESV-198AuNPs) combined radiotherapeutic and immunomodulatory functions. These nanoparticles showed a high stability in rat serum, decreased accumulation, and reduced potential for off-target effects in tissues. RESV-198AuNPs reduced pro-inflammatory and immunosuppressive cytokines (IL-1β, IL-6, IL-10, and TGF-β1) alongside the upregulation of key antitumor mediators (IL-12 and TNF-α). RESV–198AuNPs also significantly inhibited tumor growth after treatment compared to the saline control [51].

Notably, tannic acid (TA) represents a paradigmatic example of polyphenols acting beyond conventional drug carriers. Modification with TA enhanced nanoparticle stability, as demonstrated by exceptional colloidal stability that remained unchanged under various conditions. The AuNPs@TA polyphenol–metal nanoparticle (PMN) was synthesized via an in situ reduction process, then incorporated into a carboxymethyl chitosan and oxidized fucoidan hydrogel to reduce toxicity (COA hydrogels). Benefiting from the abundant polyphenolic groups of TA, the COA hydrogels exhibited significantly enhanced antioxidant activity compared with the hydrogel without AuNPs@TA PMN, effectively scavenging ROS and reactive nitrogen species (RNS). The integration of TA-based polyphenol–metal nanoparticles with PTT had significantly increased IL-10 expression, stimulated neovascularization and angiogenesis, and accelerated wound healing [52]. Moreover, in another study, small-sized TA-AuNPs (5 nm) and TA-AgNPs exhibit comparable antiviral activity, whereas at larger sizes (30 nm), only TA-AgNPs remain effective. Intranasal administration of TA-NPs in mice reduced viral titers and increased antiviral cytokines and cytotoxic cell infiltration. Moreover, TA coating mitigated Ag-induced inflammation and facilitated faster clearance, while AuNPs were more persistent in tissues, particularly in the brain, where they further contributed to immune activation [92].

In addition to AuNPs, silver nanoparticles (AgNPs) have also been used for polyphenol delivery to improve the aqueous solubility and stability of rutin, thereby enabling its application as a nano-anticoagulant with enhanced antithrombotic activity [54]. Rutin@AgNPs maintained sustained blood concentrations of rutin and significantly prolonged its circulation half-life, thereby effectively inhibiting thrombosis over 48 h, outperforming free rutin and heparin. Furthermore, they demonstrated good serum stability, hemocompatibility, and cytocompatibility.

Beyond noble metals, mineral-based inorganic systems further illustrate the versatility of polyphenol-driven nanostructures. Tannic acid–mineral nanoparticles (TMP) were developed through the self-assembly of tannic acid in an ion-rich simulated body fluid containing Ca^2+^ and PO4^3−^ aiming to achieve dual functions of osteoinduction and regulation of osteoclast maturation [53]. Leveraging the metal–phenolic network of tannic acid, TMP/Gel demonstrated excellent biocompatibility and ROS scavenging abilities, thereby modulating osteoclastogenesis and inflammatory macrophage maturation. Furthermore, in vivo testing in a mouse calvarial defect model showed significant enhancement in both the quality and quantity of newly formed bone, suggesting that these multifunctional cryogels could be effective in regulating complex bone-healing processes. Similarly, gallic acid-functionalized alumina nanoparticles and copper oxide nanorods enhance biocompatibility, reduce cytotoxicity, and amplify the inherent antioxidant activity of polyphenols [93]. The system consists of GA-functionalized copper oxide NPs (Ga@CuO) loaded with PTX. These Ga@CuO NPs were coated with K-carr and further functionalized with folic acid (FA) to enhance targeting (Ga@CuO-PTX@K-Carr/FA NPs). GA@AlOOHNPs or Ga@CuO-PTX@K-Carr/FA NPs exhibited negligible cytotoxicity via hemolysis assays, enhanced ROS scavenging capacity, and showed significantly higher anti-inflammatory and anticancer activity.

### 3.4. Other Nanoparticles

In addition to polymeric, lipid-based, and inorganic nanoparticle systems, several other nanoparticle platforms have also been explored for polyphenol delivery. One approach involves using polyphenols as both the bioactive compound and the self-carrier for nanoparticle systems. For example, Saha et al. prepared rutin nanoparticles (~200 nm in size, zeta potential −22.70 ± 2.51 mV) using an antisolvent co-precipitation technique with surfactants, such as hydroxypropyl methylcellulose (HPMC) and mannitol [56]. Compared with raw rutin, which has a particle size ranging from 1 to 200 µm, the nanoparticles showed a significant reduction in size, resulting in enhanced solubility. The nanoparticles also exhibited a sustained in vitro drug release profile (83.5 ± 8.4% over 8 days), in contrast to the rapid release of raw rutin. Another approach is to combine various types of materials, such as polymers, lipids, and inorganic components, to develop multicomponent nanoparticles. Specifically, to demonstrate the combination of polymer and inorganic components, tannic acid-modified zeolitic imidazolate framework-8 nanoparticles were loaded into an alginate-gelatin hydrogel scaffold, which showed sustained drug release and enhanced wound-healing activity [57]. Another study by Cui and colleagues developed a nanocomplex comprising the polymer, soy protein isolate, and the organic compound, glycyrrhizin, for delivering resveratrol [58]. The nanoparticles significantly enhanced the solubility of resveratrol and demonstrated pH-responsive drug release, enabling targeted delivery to the intestine. *In vitro* activity studies showed improved antioxidant properties, while in vivo experiments revealed a 5.17-fold increase in bioavailability and enhanced hepatoprotective effects against acetaminophen-induced liver injury.

## 4. Polyphenol-Based Nanoparticles for Immune Modulation

Besides acting as therapeutic agents, polyphenols can also be exploited as materials for building nanoparticles in which they function as self-therapeutic carriers or nanoplatforms for the delivery of diverse drug molecules (Table 2).

### 4.1. Self-Therapeutic Polyphenol Nanoparticles

Polyphenols can undergo self-assembly via chelation with metal ions (e.g., Fe^3+^, Zn^2+^, Cu^2+^, Mn^2+^, and Al^3+^) to generate nanoprecipitates or nanoparticles. The highly dynamic interactions between polyphenols and metal ions suggest the potential for controlled release under specific environmental conditions, thereby enhancing their applicability in targeted therapies. The adverse effects of oxidative damage on human health have driven increasing interest in potent antioxidants for immune support. The antioxidant activity of metal–polyphenol complexes is highly dependent on the type of coordinated metal ions. Gallic acid exhibited considerable antioxidant activity through chelation with Ca^2+^, Cu^2+^, Cr^3+^, and Se^6+^ [97]. Notably, complexation with Zn^2+^, involving the coordination of two gallic acid moieties per zinc ion, produced the most potent antioxidant effect, with significantly higher ABTS radical-scavenging activity than gallic acid, ascorbic acid, and Trolox [96,97]. This enhanced activity can be attributed to the synergistic ROS-scavenging effects of gallic acid and zinc ions. Based on their antioxidant properties, Zn^2+^–gallic acid nanoparticles regulated glycemic levels with negligible cytotoxicity in L6 myotubes and hepatocytes, thereby mitigating oxidative stress induced by hyperglycemia [96]. In addition, Zn^2+^–gallic acid nanoparticles also exhibited strong selective adhesion to Gram-positive bacteria through interactions between quinone groups on the nanoparticles and amino groups of teichoic acids present in Gram-positive bacterial cell walls. In a methicillin-resistant *Staphylococcus aureus* (MRSA) keratitis model, Zn^2+^–gallic acid nanoparticles enhanced bacterial clearance and tissue repair by enabling controlled Zn^2+^ release at the infection site under acidic and hypersaline conditions and by suppressing inflammation through reduced TNF-α expression [98]. Moreover, these nanoparticles also exhibited anti-cancer properties through selective cytotoxicity toward cancer cells compared with normal cells [97].

Similarly, tannic acid-based nanocomplexes fabricated via nanoprecipitation through chelation with FeCl_3_ exhibited superior radical-scavenging activity, thereby more effectively mitigating oxidative stress in human primary skin fibroblasts compared with tannic acid, ascorbic acid, and N-acetyl-L-cysteine. In an in vivo model, these tannic acid-based nanoparticles provided significant protection against oxidative stress and effectively protected *Dugesia japonica* from tert-butyl hydroperoxide-induced damage [99]. Epigallocatechin-3-gallate (EGCG) is another polyphenol with strong metal-chelating capability. Cu^2+^–EGCG nanosheets synthesized via oxidative coupling assembly for macrophage targeting exhibited good biocompatibility and negligible cytotoxicity toward RAW264.7 macrophages. In an LPS-induced inflammatory model, Cu^2+^–EGCG nanosheets outperformed free EGCG by suppressing NF-κB activation while simultaneously activating the Nrf2 pathway, leading to significant reductions in TNF-α, iNOS, IL-1β, and IL-6 levels in activated RAW264.7 cells. Furthermore, immunofluorescence analysis confirmed that Cu^2+^–EGCG promoted macrophage repolarization from the pro-inflammatory M1 phenotype toward the anti-inflammatory M2 phenotype, thereby facilitating inflammation resolution [94].

Polydopamine is a synthetic, polyphenol-like polymer enriched with catechol and quinone moieties. Owing to its phenolic-rich structure and functional similarities to naturally occurring polyphenols, polydopamine is discussed in this review as a promising material for both therapeutic applications and drug-delivery purposes. Notably, polydopamine (PDA) exhibits strong light absorption in the near-infrared (NIR) region and can act as a photothermal agent for cancer therapy. However, photothermal therapy based solely on PDA often fails to achieve sufficient tumor inhibition; therefore, combination strategies are typically required to enhance therapeutic efficacy [95]. Su and colleagues developed a nanosystem by loading EGCG onto PDA nanoparticles via Fe^3+^ chelation (EGCG@PDA), thereby integrating photothermal therapy and chemodynamic therapy. EGCG@PDA nanoparticles undergo degradation in the weakly acidic intracellular environment, leading to the release of EGCG and Fe^3+^ ions. EGCG suppresses the expression of heat shock proteins (HSPs) in cancer cells, thereby abolishing their thermoprotective effects and enhancing photothermal sensitivity. Meanwhile, the released Fe^3+^ catalyzes Fenton reactions to generate hydroxyl radicals (•OH), contributing to chemodynamic therapy–mediated cytotoxicity. Consequently, EGCG@PDA significantly reduced cell viability across multiple cancer cell lines. In vivo studies further demonstrated that EGCG@PDA nanoparticles preferentially accumulated in tumor tissues via the enhanced permeability and retention (EPR) effect, induced cancer cell apoptosis, and suppressed the expression of HSP70 and HSP90, resulting in a marked reduction in average tumor weight. Collectively, these findings indicate that EGCG@PDA represents a promising self-therapeutic nanosystem for synergistic chemodynamic therapy and enhanced photothermal therapy in cancer treatment.

### 4.2. Small Molecule Drug Delivery by Polyphenol-Based Nanoparticles

**Figure 3:**
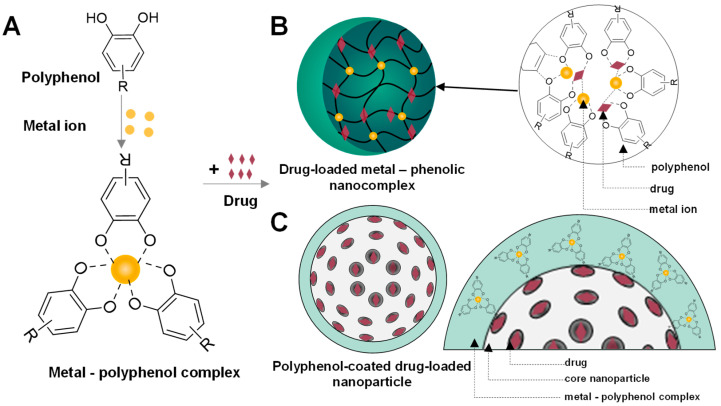
Structure of polyphenol-based nanoparticle. (**A**) Polyphenol can self-assemble through metal-ligand coordination with multivalent metal ions. (**B**) The chelation process induces nucleation and growth of metal–polyphenol complexes, which further condense into nanoparticles with tunable size, morphology, and surface characteristics. Therapeutic agents can be co-coordinated, encapsulated, intercalated during complexation, resulting in metal–polyphenol-drug nanoparticles capable with controlled release, enhanced stability, and multifunctional biomedical activity. (**C**) A metal–polyphenol complex can also be synthesized as a coating layer on the surface of drug-loaded nanocarriers to prevent burst release.

Metal–polyphenol complexes can be assembled with small molecules and various substrates through both covalent interactions (including Michael addition, Schiff base reactions, and coordination bonding), as well as noncovalent interactions (such as hydrogen bonding, π–π stacking, and electrostatic interactions). Polyphenols typically possess two or more phenolic hydroxyl groups and aromatic rings, which can be interconnected via C–C or ester bonds. Under appropriate conditions, deprotonation of the phenolic hydroxyl groups exposes oxygen donor sites, enabling coordination with metal ions and the subsequent formation of metal–polyphenol complexes [111]. The drug can be assembled in a metal–polyphenol nanocomplex or coating layer by coordination bonds, hydrogen bonds, and π–π stacking interactions (Figure 3). These metal–polyphenol nanoparticles effectively protect small-molecule drugs from rapid clearance by the reticuloendothelial system due to their nanoscale size. Moreover, they enhance therapeutic efficacy and reduce systemic side effects by enabling controlled and environment-responsive drug release.

Jin and colleagues developed a nanodrug delivery system by constructing a Fe^3+^/tannic acid (TA) metal–polyphenol network with encapsulating curcumin (Cur@Fe-TA) to improve the solubility and release profile of this therapeutic polyphenol. The therapeutic efficacy of this platform was evaluated in an in vivo dextran sulfate sodium (DSS)-induced colitis model. The results indicated that mice treated with Cur@Fe–TA nanoparticles exhibited minimal changes in colon length and morphology, comparable to those of the control group. In addition, reduced apoptosis in colonic tissues was observed compared with the untreated group, confirming the therapeutic efficacy of Cur@Fe–TA nanoparticles. Furthermore, increased levels of superoxide dismutase and glutathione, along with decreased levels of the pro-inflammatory cytokines IL-6 and IL-1β, indicated that Cur@Fe–TA nanoparticles possessed both antioxidant and anti-inflammatory activities. Therefore, this platform contributed to the regulation of oxidative stress and inflammatory responses in the gastrointestinal tract [100].

Polyphenol-based nanoparticles have also been utilized to deliver chemotherapeutic agents as a novel strategy to overcome chemotherapy resistance in lung cancer by eliminating lung microbiota. Tannin-based metal–polyphenol nanoparticles were developed for the targeted delivery of etoposide to lung tumor tissues. Notably, these nanosystems were designed by coordinating tannin with ion gallium (Ga^3+^), followed by coating with capsular polysaccharides from Streptococcus pneumoniae to mimic normal tissue components and thereby reduce immune clearance. The nanoparticles, which were degraded in the acidic microenvironment at tumor tissues, not only achieved targeted drug release but also enhanced Ga^3+^ accumulation at the lung tumor site, thereby effectively promoting pulmonary microbiota clearance by disrupting bacterial iron metabolism. In multiple mouse models of lung cancer, the nanoparticles significantly suppressed tumor growth and prolonged survival through the synergistic antibacterial effect of Ga^3+^ and the anticancer activity of etoposide [101].

Nanocomplexes synthesized via one-step self-assembly of EGCG, Cu^2+^, and diethyldithiocarbamate in an aqueous solution selectively inhibited multiple antibiotic-resistant Gram-positive bacterial strains. In contrast, the individual components used for nanoparticle synthesis showed no detectable antimicrobial activity against MRSA, even at high concentrations, highlighting the pronounced synergistic effects generated by nanoparticle assembly. Furthermore, the nanocomplexes demonstrated excellent blood biocompatibility and negligible cytotoxicity, as evaluated by hemolysis assays, acute zebrafish embryo toxicity tests, and long-term toxicity assessments in nematode models. In an infected wound model, the nanocomplexes promoted wound healing more effectively than vancomycin, evidenced by a greater reduction in wound area [102].

### 4.3. Biologic Drug-Delivery by Polyphenol-Based Nanoparticle

Polyphenols have been reported to bind to nucleic acids through hydrogen bonding and hydrophobic interactions. Thus, they can protect nucleic acids from nuclease degradation, facilitate intracellular delivery, and promote endosomal escape, thereby improving RNA interference (RNAi) efficiency [112]. Tannic acid protected siRNA from the degradation of enzymes and enhanced the uptake of siRNA into HaCaT cells to reduce the expression of NF-κB p65 and inflammatory cytokines, such as IL-1β, IL-6, TNF-α, and IL-23. Tannic acid also modulated the size of siRNA-loaded nanoparticles, thereby facilitating the transdermal delivery of siRNA for inflammation control in an imiquimod-induced psoriasis-like mouse model, as evidenced by improved skin integrity, reduced epidermal thickness, and alleviation of psoriasis-like symptoms [103].

Moreover, tannic acid served as a “molecular glue” that mediated the self-assembly of branched-DNA incorporating antisense DNA and a DNAzyme to form a nano-complex for a sequential drug release in cancer cells for treating lung cancer in BALB/L mice. Sequential drug release enabled the nanocomplexes to first liberate tannic acid within the acidic lysosomal environment and subsequently release therapeutic genes in the cytoplasm in response to glutathione/deoxyribonuclease I (GSH/DNase I), ensuring that each payload is activated at the correct intracellular location and timing. In addition to its role in nanocomplexes formation, tannic acid also exerted intrinsic therapeutic activity by inducing apoptosis in A549 cells and inhibiting their proliferation. Concurrently, the anti-sense DNA suppressed cancer cell proliferation, while the DNAzyme inhibited cancer cell migration [107].

In addition to mediating DNA assembly, tannic acid facilitated the crosslinking of Mn^2+^ and DNAzymes through metal chelation and hydrogen-bond interactions with nucleic acids to form self-assembled manganese-loaded DNAzyme nanoparticles. In acidic environments, the nanoparticles degraded, releasing Mn^2+^ and DNAzyme, which subsequently promoted •OH radical generation and mRNA cleavage. This design enhanced nanoparticle endocytosis by cancer cells, thereby overcoming the intrinsically low cellular uptake efficiency of DNAzymes. The nanoparticles exhibited higher cytotoxicity and rate of mRNA silencing than free DNAzyme. In vivo study further demonstrated that these nanoparticles significantly reduced the volumes and weights of tumors by simultaneously providing toxic •OH and suppressing gene expression through mRNA silencing [108].

To achieve more effective protection, tannic acid was combined with chitosan to form a layer-by-layer self-assembly protective coating on the surface of TNF-α siRNA–encapsulated nanoparticles. The multilayer shells composed of tannic acid and chitosan facilitated safe passage through the gastrointestinal tract, selective accumulation at inflamed colonic tissues, and efficient cellular uptake of the nanomedicine, owing to the prolonged residence time and strong cell affinity of tannic acid. Following internalization, intracellular enzymatic activity triggered the release of TNF-α siRNA, ultimately alleviating inflammation. Notably, after degradation of the coating, tannic acid was further metabolized by resident microbiota into more bioactive phenolic compounds capable of regulating gut microbiota–brain interactions, thereby improving feeding behavior in mice with inflammatory bowel disease [105].

Tannic acid can serve as an effective crosslinking agent for hydrogel synthesis, imparting excellent self-adhesive properties and tunable mechanical strength. A hydrogel incorporating antagomir-21–loaded tannic acid nanoparticles was developed for the treatment of intervertebral disc degeneration. After incorporation into the hydrogel matrix, the nanoparticles retained long-term bioactivity and exhibited a sustained release profile for more than three weeks. The gelation-after-injection property and the gradual increase in mechanical strength of the hydrogel enabled facile injection and effective filling of the narrow intervertebral disc space, facilitating localized transport of antagomir-21–loaded tannic acid nanoparticles into the nucleus pulposus tissue. Following cellular uptake by nucleus pulposus cells, antagomir-21 was released from the tannic acid nanoparticles and regulated extracellular matrix metabolic balance by inhibiting the mitogen-activated protein kinase/extracellular signal-regulated kinase (MAPK/ERK) signaling pathway. Meanwhile, tannic acid nanoparticles scavenged intracellular ROS and reduced TNF-α expression, thereby enabling the hydrogel-based gene delivery system to promote nucleus pulposus regeneration and intervertebral disc repair while delaying IDD progression [106].

Owing to the strong affinity of tannic acid for proteins on the mitochondrial outer membrane, tannic acid was co-assembled with superoxide dismutase (SOD) and catalase (CAT) to form TA–SOD–CAT nanocomplexes for targeted mitochondrial delivery of antioxidant enzymes. Consequently, these nanocomplexes reduced mitochondrial ROS levels in H_2_O_2_-damaged hepatocytes, restored mitochondrial membrane potential, and protected cells from oxidative stress. In addition, the nanocomplexes reduced signs of pyroptosis, such as cell membrane swelling, lactate dehydrogenase (LDH) leakage, and reduced expression levels of inflammatory proteins, including caspase-1, gasdermin D (GSDMD), IL-1β, and IL-18. In two pyroptosis-related liver disease models in mice, namely acetaminophen (APAP)-and lipopolysaccharide-induced acute hepatitis, TA–SOD–CAT nanocomplexes performed higher liver protection than N-acetylcysteine, proved by the decreased levels of alanine transaminase (ALT), aspartate transaminase (AST), and LDH in serum, increased the survival rate, reduced the inflammation by inhibiting IL-1β, IL-18 and decreased neutrophil infiltration into the liver [104].

### 4.4. Multiple Drug Delivery

Beyond their capacity as carriers of small-molecule therapeutics, metal–polyphenol complexes have emerged as versatile platforms that enable the simultaneous loading and coordinated delivery of multiple classes of bioactive agents or drugs via metal-ligand interactions, thereby facilitating combination therapies.

Tannic acid has been widely exploited as a polyphenol-based carrier for multiple drug delivery. Sun’s team synthesized a nanocomplex by chelating tannic acid with Fe^3+^/Mn^2+^ to co-encapsulate ovalbumin antigen and methylene blue (TA-Fe/Mn-OVA@MB NPs) for combined photodynamic therapy and photothermal therapy (PDT/PTT). Therefore, the TA-Fe/Mn-OVA@MB NPs significantly reduced cancer cell viability while exhibiting minimal toxicity to fibroblasts and red blood cells. Moreover, the TA-Fe/Mn-OVA@MB NPs could promote antigen presentation of DCs four-fold compared with OVA or Mn^2+^, activate CD8+ T cells, and promote the production of effector memory CD8+T cells, leading to the promotion of anti-tumor immune response. In vivo studies further demonstrated that TA–Fe/Mn–OVA@MB nanoparticles combined with phototherapy prolonged mouse survival beyond 60 days, highlighting their potential to combine with local phototherapy for antitumor immune activation [109].

Although dopamine is not a classical polyphenol, it shares multiple catechol-driven bioactivities with natural polyphenols, including antioxidant and anti-inflammatory effects, strong metal-chelating capability, bioadhesion, and the ability to form stimuli-responsive supramolecular nanostructures. These shared features justify the classification of dopamine as a polyphenol-like building block in nanomedicine. Li and colleagues designed and fabricated a nanoplatform derived from a synthetic polymeric polyphenol constructed from dopamine and Fe^3+^ for the one-pot co-delivery of doxorubicin (DOX) and glucose oxidase (GOD) in cancer therapy. The unique polyphenol structure endowed the nanoplatform with stimuli-responsive release of encapsulated therapeutic cargoes within the tumor microenvironment. The dissociation of the nanoparticles under intracellular acidic conditions promoted the release of DOX, GOD, and Fe^3+^. The GOD significantly accelerated the Fenton-catalyzed reaction in tumors, while DOX provided direct chemotherapy. Besides that, the use of Fe^3+^ was not only as a building unit for this supramolecular self-assembly, but also for an intracellular increased Fe^3+^ concentration that could accelerate the Fenton reaction for enhanced chemodynamic therapy. This cascade-amplified co-delivery strategy resulted in significantly enhanced cytotoxicity in vitro and superior tumor growth inhibition in vivo compared with free DOX, accompanied by pronounced tumor necrosis and minimal systemic toxicity. These findings demonstrate that polymeric polyphenol-based nanoparticles represent a promising and efficient platform for one-pot co-delivery of multiple therapeutic agents in synergistic anticancer therapy [110].

A critical comparison of nanoparticle platforms reveals distinct trade-offs in their suitability for polyphenol delivery. Organic nanoparticles, including liposomes, nanoemulsions, solid lipid nanoparticles, polymeric nanoparticles, and micelles, are widely favored due to their high biocompatibility, biodegradability, and established use in pharmaceutical formulations. Organic nanoparticles as drug carriers can help reduce the degradation of polyphenols caused by factors such as light, oxygen, pH fluctuations, temperature changes, and other harsh conditions in the gastrointestinal tract. Besides that, these nanoparticles can help overcome the limitations of polyphenols by modulating their solubility, physicochemical stability, bioavailability, and permeation, and by promoting their prolonged release over time. Despite these benefits, several challenges must be considered when developing these nanoparticles, such as their stability of biobased nanoparticles under varying environmental conditions. These can be sensitive to factors such as pH, temperature, and humidity, which can compromise their structural integrity and, consequently, their performance. Moreover, potential toxicity risks cannot be completely excluded when the degradation products of biobased nanoparticles can accumulate in tissues over time, potentially triggering inflammation or other harmful reactions. Besides that, several challenges include regulatory approval processes, large-scale production at sustainable costs, and maintaining product consistency.

In contrast, inorganic nanoparticles, such as gold and silver, are used to load polyphenols or coated with polyphenols, showing superior stability compared to organic nanoparticles. With a large mesoporous surface area and tunable physicochemical properties, these nanoparticles can combine multiple therapeutic modalities, including imaging and theragnostics. Importantly, their surfaces can strongly interact with polyphenols through coordination bonds (e.g., catechol–metal interactions), which enhances stability and preserves bioactivity. Moreover, polyphenol coating mitigated metal ion-induced inflammation and facilitated faster clearance. Several challenges associated with inorganic nanoparticles include their high accumulation in organs, leading to chronic toxicity over time due to the perpetual stimulation of the immune system, which induces an inflammatory condition. These safety concerns, coupled with unclear legislation, remain major barriers despite their functional advantages.

Metal–polyphenol networks represent a unique class of nanosystems that directly exploit the intrinsic metal-chelating and redox properties of polyphenols. The highly dynamic interactions between polyphenols and metal ions suggest the potential for controlled release under specific environmental conditions, thereby enhancing their applicability in targeted therapies, including antibacterial, anti-inflammatory, oncology, and heart-related applications. The reaction conditions are typically mild, simple synthesis procedures, and no toxic organic solvents are used. These nanoparticles can be directly assembled from metal ions and polyphenols, or via templating or seeding agents. These nanoparticles can co-deliver small-molecule and biologic drugs, including anticancer drugs and proteins with different molecular weights and isoelectric points. They can be readily loaded into NPs for various applications (e.g., biocatalysis, therapeutic delivery) by direct mixing, without surface modification, owing to polyphenols’ strong affinity for various guest molecules. The encapsulation by the polyphenol-metal network helps protect active substances, especially bioactive compounds, from degradation by enzymes or environmental factors within the body. However, their physicochemical stability is highly sensitive to environmental conditions such as pH and ionic strength, which may compromise their integrity in complex biological environments. Second, although the antioxidant capacity of these nanoparticles makes them effective in treating diseases in some cases, the instability of polyphenols during fabrication and storage can also negatively affect their druggability. Clarifying the metabolic fate of MPNs, including hepatic/renal clearance and enzymatic degradation, is crucial. Moreover, integrating therapies carries the risk of off-target effects and overlapping toxicities. Furthermore, the regulatory framework must evolve to accommodate the unique nature of MPNs and ensure rigorous, effective clinical evaluation.

Finally, polyphenols could be self-assembled into nanosystems to represent an emerging strategy in which they act not only as therapeutic agents but also as structural building blocks. Systems based on tannic acid or gallic acid exemplify this approach, offering high functional integration and, in some cases, relatively simple fabrication processes. These platforms maximize the intrinsic antioxidant and immunomodulatory properties of polyphenols while leveraging their self-assembly and redox capabilities. Nonetheless, they often suffer from limited structural control, sensitivity to oxidation, and environmental instability. As such, these systems remain at an early stage of development, with insufficient long-term safety data and a lack of established regulatory frameworks for clinical translation.

## 5. Applications of Polyphenol Nanoparticles as Immunotherapy for Various Diseases

Polyphenol nanoparticles exhibit apparently contradictory immunological effects, demonstrating either immunostimulatory or immunosuppressive activities depending on the biological context. In several studies, particularly in vaccine delivery and cancer immunotherapy, polyphenol nanoparticles have been reported to enhance both innate and adaptive immune responses. These effects include dendritic cell activation, macrophage M1 polarization, increased secretion of pro-inflammatory cytokines, and improved antigen presentation. Conversely, numerous reports highlight the immunosuppressive properties of polyphenol nanoparticles. In models of chronic inflammation or autoimmune disorders, these nanoparticles attenuate excessive immune activation and restore redox homeostasis. Such effects are largely attributed to their intrinsic antioxidant properties, including scavenging ROS, suppressing inflammatory signaling pathways, reducing cytokine secretion, and promoting M2-like macrophage polarization. These divergent outcomes are likely due to cell population, disease condition, and metal coordination.

### 5.1. Autoimmune Diseases

Autoimmune diseases are health conditions in which the immune system attacks the host’s own tissues instead of protecting them, leading to chronic inflammation and tissue damage. Autoimmune diseases can cause a wide range of symptoms, including excessive production of ROS and pro-inflammatory cytokines, leading to oxidative stress and loss of immune tolerance. Due to the combined antioxidant, anti-inflammatory, and immune-tolerogenic properties, the metal–polyphenol nanocomplexes are highlighted as potential immunomodulatory systems that can be tailored for disease-specific delivery.

Multiple sclerosis (MS) is a chronic inflammatory disease of the central nervous system leading to loss of myelin, primarily by autoimmune-mediated demyelination. In MS, Infiltration of immune cells into the central nervous system is closely associated with excessive ROS production. Excessive ROS production can lead to lipid peroxidation, DNA damage, and cell death, and plays a pivotal role in demyelination, axonal/neural injury, and modulation of blood–brain barrier (BBB) integrity. Although current treatments reduce immune-mediated inflammation and relapse frequency, they neither reverse established neurological damage nor prevent progressive neurodegeneration, which remains a major driver of long-term disability, including vision loss [113]. Given the critical contribution of redox imbalance to MS pathology, polyphenols such as resveratrol have attracted attention as redox-modulating agents. Resveratrol suppressed ROS generation via the NADPH-oxidase (NOX) complex and mitochondria, being a potential agent for reducing cytokine secretion in leukocytes of patients with MS. The nanoparticle formulation of resveratrol was developed as a potential treatment for disease models involving neurodegeneration. However, its therapeutic efficacy is restricted by pharmacokinetic limitations, including poor solubility and low bioavailability. The nanoparticles were composed of D-α-tocopherol polyethylene glycol 1000 succinate (TPGS), solutol and resveratrol and were prepared using a thin rehydration technique. The resveratrol nanoformulation overcomes these pharmacokinetic limitations, including an extended half-life. The nanoparticles not only provided neuroprotection but also increased the bioavailability of loaded resveratrol by approximately five times compared with the free polyphenol. It also demonstrated potential clinical utility by promoting retinal ganglion cell survival and limiting severe neurological dysfunction in an experimental autoimmune encephalomyelitis (EAE) model of demyelinating disease [114]. Here, immunomodulatory effects appear primarily as a secondary consequence of reduced oxidative stress, underscoring a redox-oriented therapeutic strategy. However, the EAE model does not fully reflect therapeutic efficacy in humans due to variability in disease induction, incomplete replication of MS features, species-specific immune responses, and limited clinical translatability. Therefore, these findings should be interpreted with caution when extrapolating to human MS.

In contrast to antioxidant reinforcement, the tolerogenic nanovaccine reported by Park et al. represents a shift toward active immune reprogramming by directly reshaping adaptive immune responses. A polyphenol-based tolerogenic nanovaccine composed of a core polydopamine nanoparticle and dexamethasone-loaded lipid shell was developed to treat MS. Dexamethasone on an abatacept-modified polydopamine core nanoparticle (AbaLDPN) exhibited effective tolerogenic activity, with lymph node accumulation peaking at 48 h post-administration, thereby promoting the differentiation of regulatory Treg cells. AbaLDPN-MOG induced the highest population of antigen-specific CD4+Foxp3+ Treg cells, thereby reducing the number of cytotoxic T cells and proinflammatory cytokine levels (TNF-α, IL-1β, IL-6, and IL-12). In contrast, AbaLDPN-MOG increased anti-inflammatory cytokine production and increased expression of IL-10 and transforming growth factor-β (TGF-β). Treatment with AbaLDPN-MOG prevented leukocyte infiltration and protected the brain and spinal cord against demyelination, resulting in the relief of symptoms in the EAE mouse model [115]. This platform reshaped pathogenic T-cell responses, highlighting the potential of polyphenol nanoparticles to restore immune homeostasis by targeting underlying molecular and cellular mechanisms.

Expanding this immunoregulatory framework, the tannic acid-based nanoparticles have demonstrated broader control over inflammatory signaling pathways, highlighting the capacity of polyphenol nanostructure to rebalance immune responses in autoimmune conditions. Rheumatoid arthritis is an autoimmune condition that can cause pain, swelling, and stiffness in joints. The patients using current treatments such as non-steroidal anti-inflammatory drugs (NSAIDs) or disease-modifying anti-rheumatic drugs (DMARD) to delay joint damage reported gastrointestinal and cardiovascular adverse effects. A new strategy for designing nanoparticles is to develop a drug-delivery system that simultaneously co-delivers specific amounts of self-antigens and tolerogenic drugs to dendritic cells to induce immune tolerance and treat autoimmune diseases. A recent study designed a lipid-coated nanoparticle with tannic acid as a core nanomaterial (CitDTN) with the surface modified with abatacept to yield AbaCitDTN for targeted delivery of dexamethasone and a citrullinated peptide to treat rheumatoid arthritis. Tannic acid supported AbaCitDTN to suppress NF-*κ*B activation and cleared ROS to regulate intracellular inflammatory signaling in DCs. Protein abatacept offers the possibility of blocking T cell activation and targeting DCs, which led to increased accumulation of the tolerogenic nanovaccine in the lymph node upon subcutaneous administration. AbaCitDTN promoted the number of Treg in vivo to maintain peripheral tolerance and control of arthritis [116].

Highlighting the versatility of this platform in distinct inflammatory contexts, Zhang et al. integrate RNA interference into a cascade-responsive decomposable nanocomplex based on a polyphenol-mediated framework, enabling programmable gene silencing in psoriasis. Psoriasis is a chronic, relapsing inflammatory skin disease with a strong genetic predisposition and autoimmune pathogenic traits. It is characterized by sharply demarcated reddish, scaly plaques on the skin driven by interactions between immune cells and keratinocytes, particularly involving IL-17, TNF-*α*, and IL-23. Moreover, the synergistic activity of TNF-*α* and IL-17 suggests that dual blockade may provide superior therapeutic benefit. Zhang’s team designed a functional nucleic acid nanomaterial to deliver siRNA, based on the complexation with tannic acid followed by encapsulation in the tetrahedral framework nucleic acid. Thanks to the unique structure, it has been shown to readily cross the cell membrane barrier without altering the integrity of the overall structure, thereby enhancing gene stability. Tannic acid has anti-inflammatory, proapoptotic, and antioxidant effects and may mediate the resizing of the nanocomplex to a suitable size for easy transport through the skin. Furthermore, it forms a protective layer that prevents siRNA degradation in harsh environments, such as enzymatic activity and lysosomal degradation. STT enabled the transdermal delivery of siRNA with high stability, which enabled efficient transdermal penetration and widespread distribution within skin tissue, overcoming the limitations of traditional siRNA delivery methods [103]. This strategy represents a transition from immune modulation to precise molecular regulation, highlighting the expanding design landscape of polyphenol nanoplatforms.

### 5.2. Cancer Immunotherapy

Nowadays, vaccination is considered a novel antitumor therapy due to its ability to elicit an antigen-specific adaptive immune response [117]. Unexpectedly, vaccination often fails to stimulate effective antitumor immune responses due to various issues, including the weak immune-stimulating capability of immunoadjuvants, insufficient antigen cross-presentation, and tumor immunosuppressive microenvironment (TIME) [117,118,119]. The metal–phenolic networks (MPNs) represent promising nanovaccine platforms for overcoming the limitations of current cancer vaccination strategies, owing to the inherent bioactivities of both metal ions and polyphenols. MPNs enable efficient co-delivery of antigens while leveraging the intrinsic adjuvant-like properties of metal ions, resulting in enhanced immune activation through stimulation of the cGAS-STING pathway, improved cross-presentation via endosomal escape, and reprogramming of the immunosuppressive tumor microenvironment [117,118].

**Figure 4:**
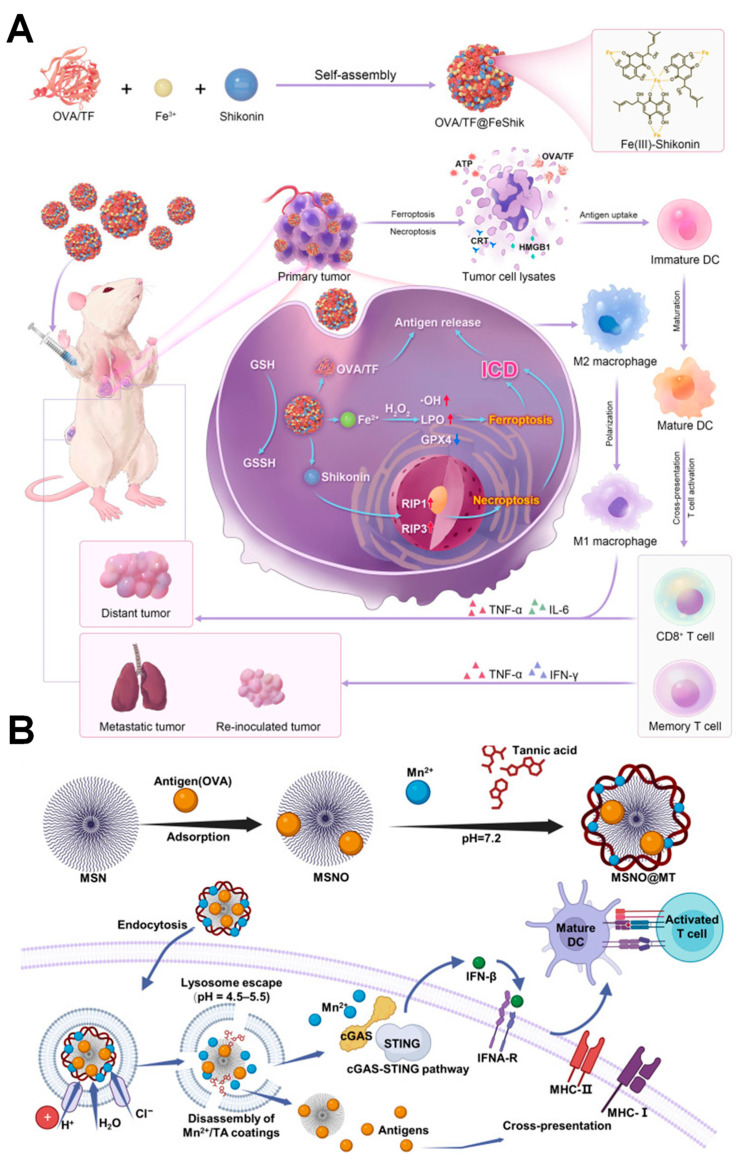
Biomedical applications of metal–polyphenol nanoparticles in cancer immunotherapy. (**A**) Following tumor accumulation and cellular uptake, FeShik nanomedicines disassemble in response to tumor microenvironmental cues, releasing Fe^2+^ and shikonin to induce immunogenic cell death via ferroptosis and necroptosis. The resulting tumor antigens and pro-inflammatory signals promote dendritic cell maturation, macrophage repolarization, and cytotoxic T cell infiltration, ultimately activating adaptive antitumor immune responses (adapted from [117] under the terms of the CC BY 4.0 license). (**B**) MSN@MT enhances antigen uptake by dendritic cells and promotes efficient cytosolic antigen delivery via lysosomal escape, while Mn^2+^ released from MSN@MT activates the cGAS–STING pathway to induce interferon-β (IFNβ) secretion, thereby facilitating antigen cross-presentation, DC maturation, and subsequent antigen-specific T cell immune responses. (Adapted from [118] under the terms of the CC BY NC 3.0 license).

Immunoadjuvants serve as indispensable components of tumor vaccines. However, many inorganic adjuvants exhibit weak immunogenicity and inadequate cellular internalization, while organic adjuvants suffer from poor circulation time. More importantly, the mono-functionality of immunoadjuvants limits their ability to fulfill the multiple functional requirements of effective antitumor immunotherapy [117]. With efforts largely focused on amplifying antitumor immunity, a multifunctional immunoadjuvant Fe^3+^-Shikonin MPNs (FeShik) capable of eliciting immunogenic cell death (ICD) was exploited [117]. In this study, metal–phenolic networks served as responsive nanocarriers for the transport and release of metal ions. Beyond their delivery role, they also functioned as immunoadjuvants that enhanced ICD. By inducing ferroptosis and necroptosis, they strengthened antitumor immune responses. FeShik loaded the model antigen ovalbumin (OVA) to form OVA@FeShik nanovaccines. The conjugation improved the drawbacks of Shikonin, thereby prolonging the blood circulation of antitumor agents, ensuring accumulation at tumor sites, and enabling responsive decomposition in the TME. Specifically, OVA@FeShik effectively elicited immunogenic cell death (ICD) in 4T1 cells through ferroptosis and necroptosis by increasing the expression of ICD-biochemical hallmarks, including high mobility group box 1 (HMGB1), calreticulin (CRT), and adenosine triphosphate (ATP). In addition, OVA@FeShik showed the highest in vivo tumor inhibition with strong polarization of M2 into M1 macrophages and enhanced infiltration of cytotoxic T lymphocytes. More importantly, OVA@FeShik exhibited a strong long-term immune memory effect against tumor metastasis and recurrence (Figure 4A).

ICD-inducing polyphenol nanovaccines represent a significant advance by enhancing tumor-associated antigen availability and initiating antitumor immune priming. Building upon this platform, the subsequent strategies have focused on promoting dendritic cell (DC) maturation and stimulating innate immune activation to achieve more robust adaptive immune responses. In particular, optimizing intracellular sensing pathways has emerged as a complementary approach to strengthen antigen presentation efficiency and T-cell priming. A MPNs based on tannic acid and manganese ions (Mn^2+^) encapsulating mesoporous silica (MSN@MT) was fabricated to serve as a nanovaccine targeting the cyclic GMP–AMP synthase–stimulator of interferon genes (cGAS-STING) pathway, promoting DC activation and antigen cross-presentation [118]. Tannic acid coordinated with Mn^2+^ through its abundant hydroxyl phenolic groups, forming a matrix system that prevents antigen degradation, significantly enhances uptake by DCs, and facilitates successful lysosomal escape. This is due to the decomposition of MSN@MT under the acidic conditions of the cellular lysosome, which causes lysosomal rupture through the influx of water molecules. Additionally, with its metal–phenolic coating, MSN@MT loading OVA (MSNO@MT) strongly activates the cGAS-STING pathway, promoting the expression of TBK1, IRF3, IFNβ, and ISGs, thus enhancing the immune response through antigen presentation and T cell activation. In vivo examination, MSNO@MT nanovaccine exhibited robust Th1 response and cytotoxic T-cell response for the clearance of cancer cells (Figure 4B).

To activate the immune system’s surveillance function, multidirectional reshaping of the tumor immune microenvironment (TIME) is a promising strategy. In a recent study, an acidity-responsive Schiff base-conjugated polyphenol-coordinated nanovaccine, Fe^3+^-TA-OVA@1-MT, was developed to reshape TIME via the polarization of tumor-associated macrophages and inhibition of indoleamine 2,3-dioxygenase (IDO), while enabling the controllable release of antigens to activate T [119]. Specifically, tannic acid forms a multi-linkage with antigen OVA via Schiff base reaction, creating the core component TA-OVA of the nanovaccine, with Fe^3+^ coordinated to prepare the Fe^3+^-TA-OVA nanosystem. Additionally, the indoleamine 2,3-dioxygenase (IDO) inhibitor 1-methyltryptophan (1-MT) was loaded to form the Fe^3+^-TA-OVA@1-MT nanovaccine, aiming to downregulate Tregs. In addition, the coordination of tannic acid and Fe^3+^ elicited a photothermal effect and tumor-specific Fenton reactions, thereby enhancing tumor immunity.

**Figure 5:**
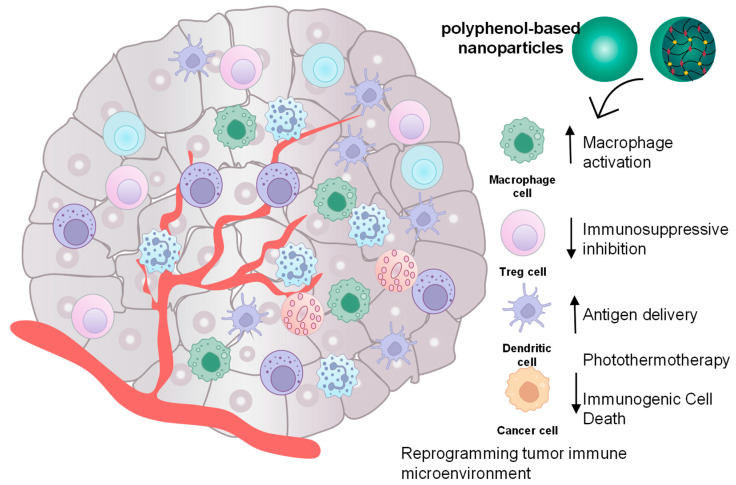
Polyphenol-based nanoparticles modulate the tumor immune microenvironment by various mechanisms. Upon accumulation within the tumor tissue, polyphenol-based nanoparticles promote macrophage activation (preferentially toward an M1-like phenotype), suppress immunosuppressive Tregs, and enhance dendritic cell-mediated antigen presentation, thereby facilitating antitumor immune activation. Photothermal therapy induces immunogenic cell death in cancer cells, further supplying tumor-associated antigens and danger signals to reinforce immune priming and amplify systemic antitumor immunity.

In short, nanocarrier-based MPNs demonstrated their amplified immune responses via multiple pathways (Figure 5).

### 5.3. Inflammatory Diseases

Polyphenol-based nanoparticles have been developed to harness the bioactivities of polyphenols, including antioxidant, anti-inflammatory, antimicrobial, and immunomodulatory properties, while addressing their inherent limitations, such as poor solubility, stability, and bioavailability. By enabling specific targeting, sustained release, and reduced systemic toxicity, polyphenol-based nanoparticles have emerged as promising therapeutic platforms. Among these platforms, polyphenol-based nanoparticles have been specifically designed to address inflammation, oxidative damage, and other pathological conditions [120,121,122].

Inflammatory bowel disease (IBD) is a chronic inflammatory disorder whose pathogenesis involves multiple factors, including genetic predisposition, environmental influences, gut microbiota dysbiosis, and immune dysregulation. Among these factors, the gut microbiome plays a critical role in IBD pathogenesis, as patients with IBD exhibit profound alterations in gut microbiota composition and structure [121]. Qinglian Hu’s team designed a nanostructured material, including *Escherichia coli Nissle* 1917 (EcN) coated with tannic acid (EcN@SApBDT-TA), to remodel the intestinal environment by regulating the redox balance, mitigating inflammation, and increasing the diversity of gut microbiota [122]. Tannic acid (TA) is a naturally derived, biologically active substance with anti-inflammatory properties and unique structures that can assemble with other components to enhance bioavailability and targeted delivery. EcN@SApBDT-TA increased the viability of probiotics and the number of them in the injured colon. Besides that, in vitro and in vivo tests showed that the SApBDT-TA coat layer also protected EcN from the attack of gastric acid when EcN@SApBDT-TA remained in the stomach for up to 6 h, thereby enabling efficient retention in the damaged colon. The intestinal mucosa of mice treated with EcN@SApBDT-TA showed excellent recovery capacity, helping to maintain intestinal homeostasis. EcN@SApBDT-TA effectively reduced ROS levels in the serum of treated mice, indicating that the administration of the drug could effectively alleviate oxidative stress in IBD mice. Collectively, EcN@SApBDT-TA represents a promising therapeutic strategy for IBD by combining probiotic delivery with intestinal microenvironment remodeling.

Zhang and colleagues reported an oral polyphenol-based nanoparticle () capable of selectively delivering drugs to inflamed sites and scavenging ROS, leading to effective inflammation control with reduced systemic side effects. Tannic acid, with strong ROS-scavenging and anti-inflammatory abilities, assembled with poly β-cyclodextrin through host–guest interaction of pyrogallol and cyclodextrin cavities to effectively encapsulate dexamethasone sodium phosphate for target delivery. The assembled polyphenol nanoparticle preserved the intrinsic antioxidant activity of pyrogallol and exhibited pH-responsive stabilization, thereby reducing the frequency of administration. Its negative surface charge promoted adhesion to inflamed colonic mucosa, supporting therapeutic efficiency and biostability. In colitis models, NPs showed anti-inflammatory effects by scavenging ROS, promoting pro-inflammatory M1 macrophages into an anti-inflammatory M2 phenotype, and inhibiting the expression of pro-inflammatory TNF-α and IL-6. The antioxidant ability of NPs was demonstrated by reducing myeloperoxidase (MPO) activity and malondialdehyde (MDA) levels, thereby suppressing oxidative stress. NPs had high biocompatibility and low cytotoxicity, with no visible histological damage or weight loss in mice. These results suggested that NPs was a promising drug-delivery system for treating inflammatory diseases [123].

Osteoarthritis (OA) is a degenerative disease characterized by cartilage erosion, synovial inflammation, and subchondral sclerosis with osteophyte formation, leading to severe pain, particularly in elderly patients [124]. In OA progression, inflammatory mediator levels increase due to the continuous production of proteases driven by proinflammatory cytokines, which stimulate chondrocytes to produce more cytokines and proteases, leading to cell senescence and death. In OA, iron and oxidative stress are accumulated, suggesting that ferroptosis plays a crucial role in OA pathogenesis [125]. EGCG, with anti-oxidant, anti-aging, and anti-inflammatory abilities, is shown to scavenge ROS and alleviate the degradation of cells induced by IL-1β, leading to a potential treatment for OA. Yu’s team developed an EGCG-based nanodrug (ES NDs) to enhance EGCG bioavailability while simultaneously delivering the antioxidant amino acid selenomethionine, which helps mitigate ferroptosis-associated OA. ES NDs showed high biocompatibility, and they were endocytosed by chondrocytes, then reduced H_2_O_2_-induced ferroptosis of chondrocytes. Compared with EGCG and SeMe, ES NDs showed a stronger reduction of ROS and reduced expression level of MDA, reducing mRNA expression levels of ferroptosis activators. ES NDs were retained in the knee joint, significantly reducing the number and volume of osteophytes in the knee joint, resulting in an increase in joint space and an improvement in subchondral bone sclerosis within the knee joint. No visible damage to articular cartilage, and the number of normal chondrocytes was observed. Therefore, ES NDs showed potential efficacy in the treatment of OA by inhibiting the inflammatory response and metabolic disorder caused by ferroptosis [126].

Periodontitis is a chronic inflammatory disease related to dysbiosis in the subgingival environment, leading to destruction of the periodontal ligament and alveolar bone. Macrophages phagocytose invading pathogens and produce pro-inflammatory and anti-inflammatory mediators, which can destroy tissues or regulate tissue homeostasis and repair them [127]. Therefore, targeting the immune microenvironment represents a promising therapeutic strategy for periodontitis. Resveratrol shows immunomodulatory effects by regulating macrophage polarization to the inflammatory M2 phenotype. However, its poor stability and low water solubility limit its clinical application. To overcome these limitations, the resveratrol-loaded liposome (Lipo-RSV) was designed to deliver resveratrol to the periodontal pocket. The Lipo-RSV led to macrophage repolarization by suppressing STAT1 phosphorylation while enhancing STAT3 activation, thereby inhibiting NF-κB signaling and reducing levels of pro-inflammatory cytokines (IL-1β, IL-6, TNF-α, and IL-12). The findings demonstrated that Lipo-RSV addresses the limitations of resveratrol and enhances its therapeutic efficacy. It also exhibited anti-inflammatory and anti-resorptive effects comparable to those of ibuprofen. In summary, Lipo-RSV could be a promising alternative to antibiotics for periodontitis treatment [36].

Building on strategies to modulate the immune microenvironment for periodontitis therapy, a multifunctional injectable hydrogel has been developed for localized periodontal treatment. The well-known antioxidant natural polyphenol, epigallocatechin gallate (EGCG), was coordinated with Cu^2+^ ions to enhance its bioavailability and stability. Subsequently, the resulting metal–phenol coordination complex was incorporated into a gelatin–Laponite matrix to form a uniform hydrogel capable of localized delivery to the periodontal pocket. This Laponite/gelatin-based hydrogel effectively addressed multiple pathogenic mechanisms of periodontitis, including bacterial infection, oxidative stress, and immune dysregulation. Both in vitro and in vivo studies demonstrated that the hydrogel not only inhibited *Porphyromonas gingivalis* effectively but also induced macrophage polarization from the pro-inflammatory M1 phenotype to the anti-inflammatory M2 phenotype. In addition, it exhibited excellent biocompatibility with mouse monocyte-macrophage leukemia cells (RAW264.7) and bone marrow mesenchymal stem cells (BMSCs), while promoting periodontal bone defect repair. Overall, these synergistic effects suggest that the EGCG–Cu coordinated Laponite/gelatin hydrogel represents a highly promising platform for localized periodontal tissue repair and regeneration [128].

Building on the same concept of coordinating EGCG with metal ions to overcome the limitations associated with its direct use, similar strategies have been applied to diabetic wound treatment, another chronic inflammatory condition sharing pathological similarities with periodontitis. In one such system, the EGCG−Zn complex was incorporated into quaternized chitosan (QCS) through electrostatic interaction between the cationic QCS and the anionic EGCG–Zn complex, thereby facilitating the self-assembly of stable nanoparticles. The resulting EGCG−Zn−QCS NPs exhibited excellent stability, dispersibility, and sustained-release behavior of EGCG. Importantly, the material demonstrated outstanding biocompatibility both in vitro and in vivo. Compared with free EGCG or EGCG–Zn complexes alone, the EGCG–Zn–QCS system significantly enhanced diabetic wound closure by reducing bacterial infection, alleviating oxidative stress, and modulating immune responses—notably promoting macrophage polarization from the pro-inflammatory M1 phenotype to the anti-inflammatory M2 phenotype, decreasing levels of inflammatory cytokines (TNF-α, IL-1β, and IL-6), and increasing the production of anti-inflammatory cytokine IL-10. Histological analysis further confirmed enhanced collagen deposition and vascularization, indicating improved tissue regeneration through upregulation of key growth and angiogenesis-related factors. This multifunctional system highlighted the potential of polyphenol-metal coordinated nanoparticles for diabetic wound healing and held substantial potential for broader clinical applications in inflammation-related tissue repair [129].

Polyphenols are also incorporated into biomaterials to significantly improve their adhesion to the skin, mucous membranes, and other tissues, and are used for the treatment of oral ulcers. Tannic acid, with antioxidant and immunoregulatory properties, accelerates oral ulcer healing. Moreover, through dynamic covalent bonds with exposed amino and sulfhydryl groups on the wound surface, TA also helps increase particle retention time, thereby enhancing the adhesion properties of materials. The nanocarriers PGA/TA-NPs were made of doxycycline hydrochloride-loaded polyglutamic acid/TA nanoparticles to enable controlled anti-bacterial drug release with strong adhesion to ulcer tissue. They helped to better inhibit bacterial invasion at the ulcer site and reduce excessive inflammatory responses. The PGA/TA-NPs showed strong ROS scavenging in RAW264.7 cells due to the easily oxidized hydroxyl phenolic group of TA and the nano’s smaller particle size, resulting in a larger specific surface area and a higher probability of contact with macrophages. Besides that, they also had antibacterial properties against both negative- and positive-gram bacteria, thanks to doxycycline hydrochloride (DCH). The PGA/TA NPs exhibited lower toxic effects than free DCH in both L929 and RAW 264.7 cells. In the oral ulcer mouse model, PGA/TA-NPs demonstrated prolonged retention at the ulcer site and accelerated wound healing rate within 6 days. Furthermore, PGA/TA-NPs promoted the differentiation of inflammatory M1 macrophages into an anti-inflammatory M2 phenotype. Therefore, through the synergistic effects of the drug and the polyphenol-based nanocarrier, an integrated therapeutic effect combining antibacterial activity, immune regulation, and tissue repair could be achieved for treating oral ulcer wounds [130].

Neuroinflammation plays a pivotal role in the progression of neurodegenerative diseases such as Alzheimer’s and Parkinson’s disease, largely driven by the overactivation of microglia toward the pro-inflammatory M1 phenotype. A polydopamine nanoparticle (PDNP)-based approach has been explored as a potential immunomodulatory nanoplatform capable of attenuating excessive microglial activation. These nanoparticles were prepared via the oxidative self-polymerization of dopamine in an alkaline aqueous ethanol solution initiated by ammonium hydroxide, yielding uniform and stable PDNPs with excellent biocompatibility and minimal cytotoxicity toward human microglial clone 3 (HMC3) cells. Upon stimulation with IFN-γ to induce an inflammatory response, the PDNPs were efficiently internalized by microglia, effectively reducing intracellular ROS accumulation, suppressing the expression of M1 surface markers (CD40, CD86), and downregulating the secretion of pro-inflammatory cytokines (IL-6, IL-8, TNF-α). Collectively, these results demonstrate that PDNPs can alleviate oxidative stress and inhibit microglial polarization toward a pro-inflammatory state. These results demonstrated that PDNPs are potential candidates for modulating microglia-mediated neuroinflammation and mitigating neurodegenerative progression; however, the current evidence was limited to in vitro models, and further in vivo studies are required to confirm their therapeutic efficacy [131].

Yuan prepared natural polyphenol-metformin nanoparticles with remarkable pH responsiveness and antioxidative and anti-inflammatory properties for spinal cord regeneration. Spinal cord injury induces oxidative stress and neuroinflammation, which can lead to neuronal loss, demyelination, and axonal degeneration. This unfavorable microenvironment around the injury site hindered stem cell survival and directed their differentiation predominantly into astrocytes rather than neurons or oligodendrocytes, which could decrease neural circuit reorganization and the conductivity of residual neural connections. Nanoparticles of EGCG and metformin improved the microenvironment around the injury site by activating neural stem cells and facilitating neurogenesis. Furthermore, these nanoparticles regulated the differentiation of activated neural stem cells into neurons and oligodendrocytes, effectively repairing injured nerve structures and promoting functional recovery in experimental rat models [132].

Bone regeneration is influenced by multiple local and systemic factors, including macrophage polarization: the M1 phenotype inhibits bone repair, whereas the M2 phenotype promotes bone repair and regeneration. Byun’s team developed a composite cryogel (TMP gel) containing a mineral metal ion (Ca^2+^, PO_4_^3−^), tannic acid, and gelatin to reduce inflammation and modulate osteoclastogenesis, thereby enhancing bone tissue regeneration. Inorganic mineral materials (Ca^2+^, PO4^3−^) have biological activity due to their chemical similarity to calcified natural bone, enhancing osteogenesis. TA has catechol and galloyl groups and can interact with metal ions to form metal polyphenol nanoparticles through metal chelation. Their disassembly in a high-pH environment led to localized ion release, which plays an important role in inducing the desired osteoinductive effect. Besides that, TA can effectively clear ROS and regulate macrophage maturation and inflammation during osteoclastogenesis. Treatment with TMP/Gel increased mRNA levels of osteogenic genes, stimulating osteogenesis and inhibiting osteoclast maturation. TMP/Gel showed anti-inflammatory effects by decreasing the expression of both pro-inflammatory and osteoclastogenic genes, driven by the synergistic effect of TA and mineral metal ions. In a mouse calvarial defect model, ICR mice treated with TMP/Gel showed a large area of regenerated bone and a higher bone tissue volume. These findings indicated that TMP/Gel could be a promising biomaterial for bone regeneration [53]. However, their translational relevance remains limited because of key anatomical and physiological differences between mouse calvarial defect models and human bone, particularly in terms of biomechanical loading and ossification processes [133].

Ischemic heart disease (IHD) results from coronary artery occlusion, leading to myocardial hypoxia due to reduced blood supply. IHD increases neutrophils and macrophages in the inflamed myocardium, leading to persistent cardiac inflammation. Anti-inflammation treatments do not eliminate free radicals, leading to failure to break the inflammation cycle and reduce cardiac damage. Liu and colleagues developed a Fe-Cur@TA nanozyme, generated by coordinating Fe^3+^ with the anti-inflammatory drug curcumin (Cur) and further modifying with tannic acid (TA), for cardiac-targeted anti-inflammatory therapy through synergistic effects of anti-inflammatory and antioxidant activities. Tannic acid had a high affinity with cardiac elastin and collagen and promoted the accumulation of Fe Cur@TA in cardiac tissue rather than blood vessels or other tissues. TA also effectively enhanced Fe-Cur@TA nanozyme cellular uptake. The increased cellular uptake of Fe-Cur@TA could enhance curcumin’s anti-inflammation by regulating macrophage polarization into M2 macrophages and decreasing the secretion of inflammatory cytokines (TNF-*α* and IL-6), not only in vitro but also in vivo. Moreover, Fe-Cur@TA promoted cardiac remodeling after ischemic injury and improved cardiac function in both MI mice and MI Beagle Dogs by reducing infarct size, promoting blood vessel regeneration, and stimulating cardiomyocyte proliferation. In conclusion, tannic acid significantly improved the uptake efficiency of Fe-Cur nanozyme in cardiac tissue and enhanced the efficacy of the anti-inflammatory and free radicals scavenging properties of the nanozymes [134].

Atopic dermatitis (AD) is the most common chronic inflammatory skin disease, characterized by genetic predisposition, epidermal barrier disruption, and immune dysregulation. The progression of AD is associated with excessive activation of specific T-cell subsets, predominantly T-helper 2 (Th2) responses, along with Th1, Th22, and Th17 pathways, resulting in elevated inflammatory cytokine production and disruption of the skin barrier. This condition is exacerbated by high levels of the mobility group box 1 (HMGB1) protein, which impair epidermal growth and stratum corneum formation. Resveratrol was shown to significantly downregulate HMGB1 expression and reduce the levels of inflammatory mediators. To overcome resveratrol’s poor oral bioavailability, resveratrol-loaded nanoemulsions were developed to enhance its penetration through the stratum corneum. Ex vivo permeation and retention studies demonstrated significantly higher skin retention of resveratrol and improved skin structure without signs of skin irritation. Furthermore, Western blotting and reverse transcription polymerase chain reaction (RT-PCR) analyses revealed reduced expression of HMGB1 and pro-inflammatory cytokines in an AD mouse model. These findings indicated that resveratrol-loaded nanoemulsions enhanced permeation, increased retention of RES in mouse skin, and improved the anti-inflammatory potential of RES in treating AD [37].

**Figure 6:**
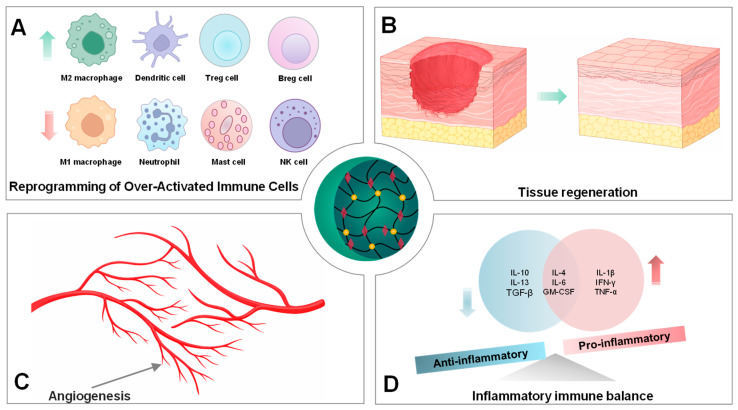
Immune regulatory activities of polyphenol nanoparticles. (**A**) Downregulation of overactivated inflammatory immune cells while enhancing regulatory and anti-inflammatory cell subsets. (**B**) Promotion of tissue healing through antioxidative activity, enhanced cellular repair, and extracellular matrix remodeling. (**C**) Modulation of vascular growth to support tissue perfusion and regenerative outcomes. (**D**) Restoration of immune homeostasis under inflammatory conditions.

In summary, polyphenol nanoparticles exert immunoregulatory effects by suppressing excessive inflammatory immune responses while promoting regulatory and anti-inflammatory cell populations, enhancing antioxidative-mediated tissue repair and extracellular matrix remodeling, modulating angiogenesis to support tissue perfusion, and ultimately restoring immune homeostasis under inflammatory conditions (Figure 6).

### 5.4. Other Diseases

Viral infections have complex pathogenesis, driven by viral replication and the host response to infection (e.g., inflammation and oxidative stress). Viral infections disrupt the body’s antioxidant defenses, leading to inflammation and oxidative damage. Polyphenols, owing to their antiviral and antioxidant activities, can enhance immune and antioxidant defense systems and, thus, represent promising agents for preventing infection onset or limiting viral disease progression. Polyphenols exert anti-inflammatory effects by suppressing the gene expression of pro-inflammatory cytokines, including IL-1β, IL-6, and IL-12p35, thereby modulating host immune responses during viral infection [135]

Among respiratory infections, human respiratory syncytial virus (RSV) is the etiological agent causing respiratory diseases in children and the elderly. Directly inactivating the virus before it enters host cells could be a potential treatment for this disease. With antioxidant effects, resveratrol has been reported to combat severe respiratory infections, particularly RSV. Resveratrol disrupted the early stages of RSV infection by engaging with viral attachment factors, such as heparan sulfate proteoglycans (HSPGs), thereby impeding viral receptor binding, inhibiting expression of the pro-inflammatory cytokine, reducing viral load in the lungs, and increasing neutrophils in RSV-infected mice [136]. In a previous study, resveratrol nanoparticles exhibited antiviral activities by reducing pro-inflammatory cytokine production and inhibiting RSV replication. These nanoparticles improved the pulmonary microenvironment and reduced RSV viral load in RSV-infected mice, thereby providing a promising new strategy for treating viral pneumonia [137]. To enhance solubility and antiviral efficacy, a dendrimer-based system against human immunodeficiency virus (HIV) was developed by conjugating curcumin to a generation-2 PEG–citrate dendrimer via an ester linkage, thereby significantly reducing the cytotoxicity of the curcumin–dendrimer conjugate. Curcumin inhibited the HIV protease activity as well as the trans-activator protein Tat, leading to the inhibition of HIV-1 replication. These findings indicated that curcumin dendrimers could have a high potential against this virus [138].

In addition, polyphenols have been shown to modulate immune-mediated respiratory disorders. Allergic rhinitis (AR) is a type-I hypersensitivity disease characterized by allergen-induced immunoglobulin E (IgE)-mediated activation of mast cells and basophils, leading to the release of histamine, TNF-α, IL-4, and IFN-γ, and recruitment of inflammatory cells that perpetuate the inflammatory response. EGCG was demonstrated to suppress the release of β-hexosaminidase, IL-4, and tumor necrosis factor-α from the IgE/antigen-stimulated mast cell model, suggesting its potential as an alternative treatment for AR. A nanocarrier system based on EGCG-loaded polycyclodextrin was developed to control the drug release profile and improve the EGCG bioavailability by incorporating thiolated chitosan, which enhances the mucosal adhesion through covalent bonding between sulfhydryl groups of thiolated chitosan (TCS) and cysteine residues of mucins on the mucosal surface. The nanosupramolecular delivery system composed of β-cyclodextrin supramolecular polymer (PCD), thiolated chitosan (TCS), and natural polyphenol epigallocatechin gallate (EGCG) (TCS/PCD@EGCG) exhibited hemocompatibility, low toxicity, and pronounced antioxidant and antibacterial effects, and effective ROS scavenging. TCS/PCD@EGCG consistently inhibited mast cell activity and histamine release while increasing the expression of an anti-allergic cytokine, thereby alleviating rhinitis symptoms compared with budesonide. In addition, TCS/PCD@EGCG reduced the frequency of administration, as shown by the degree of relief of rhinitis symptoms, which was significantly superior to the budesonide group, on which the AR mouse intermittent administration model was reconstructed [139].

## 6. Challenges and Perspectives

Although polyphenol nanoparticles have been extensively investigated for their potential in immune regulation and disease treatment, several challenges continue to impede their clinical translation. Key obstacles include long-term toxicity, limited biocompatibility, and difficulties with large-scale implementation, particularly in ensuring reproducibility, scalability, and regulatory compliance.

In general, polyphenols exhibit a favorable safety profile following oral administration, with high-tolerated doses reported in preclinical studies. Clinical evidence further supports the therapeutic relevance of polyphenols, with quercetin reducing inflammation and oxidative stress in patients with chronic obstructive pulmonary disease, and epigallocatechin gallate (EGCG) showing potential benefits in interstitial pneumonia in early-phase trials; however, these findings are primarily limited to free compounds rather than nanoformulations [140,141]. Preclinical studies have reported high-tolerated oral doses for compounds such as gallic acid (5 g/kg), curcumin (2–5 g/kg), quercetin (6 g/kg), and kaempferol (8 g/kg); however, their safety following intravenous administration remains incompletely characterized [142]. However, the safety of polyphenols following parenteral administration remains uncertain. Of note, polyphenols, when formulated into nanomaterials for therapeutic applications, may alter pharmacokinetic pathways, including absorption, distribution, metabolism, and excretion. This can result in unpredictable retention profiles or accumulation of these nanomaterials in specific organs compared with their polyphenol precursors. Therefore, the safety profile of polyphenol nanoplatforms should not be assessed solely based on that of free molecular polyphenols. Instead, they should be considered as new bioactive entities with specific physicochemical and biological properties, necessitating independent toxicological evaluation.

Polyphenols can interact with various biological components, including proteins, lipids, and nucleic acids [104,107,109]. Tannic acid is known to bind to and alter the structural conformation of fibrinogen, as well as destabilize red blood cell membranes, leading to changes in blood coagulation time and inducing hemolysis in a concentration-dependent manner [143]. The long-term toxicity of polyphenol-based nanoparticles is strongly influenced by their biodegradability. In general, polyphenol delivery systems derived from organic-based nanoplatforms, such as lipid-based nanoparticles and biodegradable polymeric nanoparticles, tend to degrade rapidly. In contrast, polyphenol-modified inorganic nanoparticles, including silver, gold, and metal oxide nanoparticles (e.g., CuO and ZnO), may pose safety concerns due to their limited biodegradability. These inorganic systems often require more rigorous evaluation of metal-related toxicity, particularly with respect to long-term retention, accumulation, and potential chronic biological effects.

Various therapeutic effects of polyphenol nanoparticles have been examined via intravenous injection. However, interaction with plasma proteins and the in vivo fate of these polyphenol nanoparticles have not been elucidated. Indeed, whether in the soluble forms or as nanoparticles, polyphenols can interact with plasma proteins absorbed on their surface, a process known as opsonization [144]. Opsonization of polyphenol nanoparticles may facilitate recognition by the immune system and, thus, promote clearance from the body. In addition, opsonization may also hinder the active surface of polyphenol nanoparticles and, thus, compromise the potential cellular binding effects [145]. In addition, this opsonization possibly increases the risk of complement activation–related pseudoallergy (CARPA) when these polyphenol nanoparticles are repeatedly injected. This phenomenon has been reported during liposome treatment, which resulted in side effects ranging from mild to severe, such as respiratory and/or cardiac arrest. Cautious studies on sensitive animals, such as the pig model, should be conducted to fully evaluate the latent reactions [146]. PEG coating has been introduced in several studies to prevent opsonization [147]. Using this approach, factors influencing the steric effect of PEG, including polymer chain density, molecular weight, and grafting methods (covalent or non-covalent), should be systematically optimized.

Clinical translation of polyphenol nanoparticles may face various barriers, including limited preclinical data and limited biological relevance. Indeed, most current studies use 2D-cell culture models of either single immune cells or cancer cells to evaluate the efficacy, which do not reestablish the complexities of a 3D immunological environment as well as the cell–cell interactions. In addition, the toxicity and efficacy data in mice do not resemble the activities of polyphenol nanoparticles in humans, where there are significant species-specific differences, such as metabolism and immune system activities (e.g., complement systems, TLR patterns, magnitude of cytokine response). These challenges would lead to unpredictable effects of nanoparticles on human systems.

Metal–polyphenol nanocomplexes have been explored in many studies as versatile drug delivery platforms via various administration routes, including intravenous injection [95,98,101]. Administration of these formulations may raise concerns about metal-related toxicity, as a small amount of metal ions is released from the formulations during circulation and accumulates in the body over time. Of note, the activities of metal ions depend on the ion type and their chelating polyphenol ligands [97,148]. Biologically prevalent metal ions such as Zn^2+^, Fe^3+^, Ca^2+^, and Cu^2+^ generally exhibit higher physiological tolerance, whereas less abundant or redox-sensitive ions, including Se^4+^, Ga^3+^, and Cr^3+^, may pose greater toxicological risks. Moreover, the chelation strength, coordination geometry, and ligand-exchange kinetics of different polyphenols can significantly influence the in vivo metal-ion release profiles. Consequently, comprehensive toxicological evaluation of metal–polyphenol nanomedicines must account for both components of the complex. Accurate quantification of metal ion content, release kinetics, and long-term biodistribution is essential to distinguish polyphenol-mediated biological effects from metal-induced toxicity. Without such systematic assessment, the translational potential of metal–polyphenol nanocomplexes remains constrained by uncertainties related to safety and regulatory acceptance.

Compatibility of polyphenols with other excipients, particularly active pharmaceutical ingredients, requires careful consideration during formulation development. In alkaline conditions and/or in the presence of oxygen, polyphenols undergo auto-oxidation and become quinones, which act as pro-oxidants [149,150]. Therefore, encapsulating drugs with amine or sulfhydryl groups in polyphenol-based nanoparticles could accelerate their degradation. In addition, polyphenol nanoparticles involving metal chelation, such as Fe^3+^ or Cu^2+^, may undergo the Fenton reaction and generate ROS, potentially destabilizing sensitive drugs [95,151]. Adding reducing agents to the formulation to prevent polyphenol oxidation may be necessary to improve drug stability. The auto-oxidation process also raises concerns regarding the stability of polyphenols in storage, especially in liquid form. Formulating polyphenol nanoparticles in freeze-dried form could improve their long-term stability.

Polyphenol nanoparticles composed of multiple components pose significant technical and quality control challenges for scalable manufacturing. Formulations of these platforms involve multiple interacting factors, such as polyphenol concentration, stabilizer concentration, pH, ionic strength, mixing rate, etc. Small variations in these factors can alter particle size, size homogeneity, surface properties, drug loading capacity, and release profile, thereby affecting the batch-to-batch reproducibility under Good Manufacturing Practice conditions. From a regulatory perspective, these polyphenol nanoplatforms face additional hurdles regarding structural complexity, detailed physicochemical characterization, and validated analytical methods. Microfluidic platform-based manufacturing can provide control over mixing kinetics, flow rate, and shear stress to address the challenges in bath reproducibility.

Recent studies have suggested that polyphenols have a positive impact on approved immunotherapy approaches, such as programmed cell death protein 1/programmed death-ligand 1 (PD-1/PD-L1) immune checkpoint blockade therapy. From a translational perspective, a rational schedule may involve preconditioning the tumor microenvironment with polyphenol nanoparticles prior to checkpoint inhibitor administration to reduce PD-L1 expression and inflammatory immunosuppressive signaling before T-cell activation. Apigenin, luteolin, silibinin, and hesperidin have been reported to inhibit STAT-3 signaling, while EGCG and hesperidin inhibit STAT1 signaling pathways, contributing to the suppression of PD-L1 expression. Gallic acid regulates the p53-miR-34a pathway and, thus, downregulates PD-L1 mRNA expression in cancer cells [152]. Formulating these polyphenols into nano-delivery systems may overcome poor solubility and non-specific distribution, thus improving the adjuvant effect when combined with PD-1/PD-L1 blockade therapy. In addition, polyphenol nanoparticles may mitigate exhaustion pathways and oxidative stress within the tumor microenvironment while potentially improving CAR-T persistence and cytotoxicity [153].

Cell-based immunotherapies may also benefit from polyphenol nanoparticles. In recent years, cell-surface-engineered nanoparticle delivery systems have been developed by integrating functional nanoparticles onto therapeutic cells [154]. These nanoparticle-modified cells are subsequently re-infused into patients, where they function not only as active therapeutic cells but also as mobile delivery platforms for the attached drug-loaded nanoparticles. Gallic acid co-administered with anti-CD19 chimeric antigen receptor T (CAR-T) cells enhanced treatment outcomes by increasing proliferation, cytokine production, and the killing effect of CAR-T cells [155]. Therefore, cell-surface-engineered nanoparticle delivery systems can be extended to gallic acid-based nanoparticles, which are engineered to bind to the surface of CAR-T cells ex vivo. This may enhance the effect of gallic acid with an optimized release pattern and pharmacokinetics through an appropriate nanoplatform design. CAR-T cells have also been genetically engineered to express resveratrol-inducible systems that control their activation and suppression, thereby improving the safety of CAR-T cell therapy. However, this strategy requires a controlled administration of external resveratrol [156]. Magnetically responsive nanoparticle carrying resveratrol, such as iron oxide nanoparticles, attached to this CAR-T cell system could be exploited to control resveratrol release and thus conveniently regulate CAR-T cell activities via an external magnetic device.

Given the urgent need for vaccine development to prevent infectious diseases such as COVID-19, mRNA vaccines have gained great attention due to their rapid scalability. The translation of mRNA vaccines into clinical use would not have been feasible without the profound development of lipid nanocarriers. However, these platforms still face challenges such as endosomal trapping, which reduces the efficacy of mRNA vaccines. Polyphenol-based nanoparticles could improve the design of mRNA vaccine carriers. A computational study has developed structurally diverse polyphenol nanoparticle platforms (PARCELs), which surpass lipid nanoparticles in terms of endosomal escape efficiency [157]. With the growth of artificial intelligence, understanding the structure of polyphenols and their impact on delivery efficacy parameters would guide the optimal design of mRNA vaccine formulations.

These challenges highlight the need to move beyond proof-of-concept studies toward rational, mechanism-guided design principles for polyphenol-based immunonanomedicine. Progress in this field will depend on the integration of immune biology, materials science, and nanotechnology to achieve precise immune modulation while enhancing translational potential.

## 7. Conclusions

Polyphenols exhibited potential immunomodulatory and therapeutic activities. However, their clinical use is still limited by poor solubility, low bioavailability, and rapid metabolism. Nanocarrier-based delivery systems have emerged as effective strategies to overcome these challenges by enhancing the solubility, targeting ability, and pharmacological performance of polyphenols. Moreover, recent development of polyphenol-based nanocarriers demonstrated promising applications in drug delivery due to the intrinsic effect of polyphenol as an immunomodulatory agent, besides the multifunctional building blocks for carrier formation. Despite the advanced outcome of polyphenol nanoparticles in preclinical tests, the challenges persist, such as rational design of multicomponent nanoparticles, in vivo fate of polyphenol-based carriers, and long-term safety of new materials. The potential of polyphenols could be extended by integrating with immunotherapeutics, which could offer a synergistic effect and advance the development of clinically translatable immunoregulatory nanoplatforms.

## Figures and Tables

**Table 1:** Applications of polyphenol nanoparticles in immunomodulatory therapeutics.

Nanoparticles Type	Carrier Composition	Delivered Polyphenol	Activities	Ref.
**Lipid-based nanoparticles**	Liposome: Soybean lecithin, cholesterol, and DSPE–mPEG 2000	Curcumin	Enhance tumor cytotoxicity in HT-29, HCT-116, and HGC-27 cell lines	[35]
	Liposome: egg yolk lecithin (PC-98T), cholesterol, and DSPE-mPEG2000,	Resveratrol	Re-educate the inflammatory macrophages from M1- to M2-like phenotype. Reduce the pro-inflammatory cytokines Alleviate periodontitis in a ligature-induced periodontitis mouse model	[36]
	Nanoemulgel:SEPINEO™ P 600 gel base and propylene glycol		Improve the atopic dermatitis skin condition	[37]
	Nanoemulsion: Hyaluronic acid-poly(glyceryl)10-stearate		Enhance bioaccessibility of resveratrol in the system	[38]
	Solid lipid nanoparticle: Stearic acid, lecithin, and D-α-Tocopheryl polyethylene glycol 1000 succinate		Inhibit cell migration Induce mitochondrial dysfunction Improve tumor treatment efficiency by inducing apoptosis	[39]
	Solid lipid nanoparticle: Phospholipon^®^ 80H	Rutin	Enhance antioxidant effects in human glioblastoma astrocytoma (U373) cells	[40]
**Polymeric nanoparticles**	Natural polymer-based systems: Chitosan, sodium tripolyphosphate, β-Cyclodextrin	Curcumin	Decrease miR-221, miR-222, and β-catenin expression and increase WIF1 expression in breast cancer cell lines.	[41]
	Natural polymer-based systems: Zein, carboxymethylated short-chain amylose		Increase the scavenging capacity of DPPH• free radical	[42]
	Natural polymer-based systems: Chitosan	Resveratrol	Enhance cytotoxicity against MCF7 and SKBr3 breast cancer cell lines	[43]
	Natural polymer-based systems: Chitosan, pluronic F127, and PVA	Urolithin B	Exhibit selective cytotoxicity toward pancreatic cancer cells and significant antibacterial activity against both Gram-positive and Gram-negative bacteria.	[44]
	Natural polymer-based systems: Chitosan	Rutin	Improve neurobehavioral activity, reduce infarct volume, and enhance brain-targeting efficiency in a cerebral ischemia rat model.	[45]
	Natural polymer-based systems: Zein		Enhance antioxidant activity	[46]
	Synthetic polymer-based systems: Poly(lactic-co-glycolic acid) (PLGA)	Tannic acid	Reduce T-cell infiltration within grafts, decrease rejection grades, and markedly extend graft survival time in a heart transplantation model	[47]
	Synthetic polymer-based systems: PLGA	Rutin	Reduce hepatic nodules and enhance antioxidant enzyme levels	[48]
	Synthetic polymer-based systems: Eudragit S100		Enhance cytotoxicity against human colon cancer cells	[49]
**Inorganic nanoparticles**	Gold nanoparticles, Zn^2+^	EGCG	Inhibit peptide self-association and fibrillization associated with amyloid aggregation	[50]
	Gold nanoparticles	Resveratrol	Promote anti-tumor immunity in PC-3 tumor-bearing SCID mice	[51]
	Gold nanoparticles, carboxymethyl chitosan, oxidized fucoidan hydrogel	Tannic acid	Promote postoperative melanoma suppression and skin regeneration in vivo.	[52]
	Mineral nanoparticles: Ca^2+^, Mg^2+^, Na^+^, K^+^, and PO_4_^3−^, gelatin-based cryogel		Reduce inflammation and regulate osteoclast maturation	[53]
	Silver nanoparticles	Rutin	Prolong the activated partial thromboplastin time and prothrombin time and inhibit thrombosis over 48 h period	[54]
	Copper oxide nanoparticles, kappa-carrageenan, folic acid	Gallic acid	Enhance anticancer activity in breast cell lines	[55]
**Other nanoparticles**	Self-carrier: Rutin, hydroxypropyl methylcellulose and mannitol	Rutin	Exhibit a sustained drug release profile in vitro	[56]
	Multicomponent nanoparticles: zeolitic imidazolate framework-8, alginate-gelatin hydrogel	Tannic acid	Enhance wound healing activity	[57]
	Combination of polymer and organic materials: Soy protein isolate, glycyrrhizin	Resveratrol	Enhance hepatoprotective effect against overdose acetaminophen-induced liver injury	[58]

**Table 2:** Applications of Polyphenol-Based Nanoparticles in Modulating Immune Responses.

Cargo Type	Polyphenol	Carrier Composition	Drug(s)	Size	Activities	Ref.
**Non-cargo**	EGCG	EGCG, Cu^2+^	-	218 nm	Enhance antioxidant and anti-inflammation properties	[94]
		EGCG, Fe^3+^	Polydopamine	170.2 ± 0.184 nm	Induce cancer cell apoptosis Reduce mean tumor weight	[95]
	Gallic acid	Gallic acid, Zn^2+^	-	no	Prevent oxidative stress damage by hyperglycaemia	[96]
		Gallic acid, metal ions (Ca(II), Cu(II), Cr(III), Zn(II) and Se(VI))	-	4.90–93.87 nm	Induce selective cytotoxicity against cancer cells over normal cells	[97]
		Gallic acid, Zn^2+^	-	20 nm	Accelerate bacterial clearance Promote faster tissue healing Reduce inflammatory response	[98]
	Tannic acid	Tannic acid, Fe^3+^, DSPE-PEG	-	201 ± 4 nm	Reduce oxidative stress in human primary skin fibroblasts	[99]
**Small molecule**	Tannic acid	Tannic acid, Fe^3+^	Curcumin	191.7 nm	Regulate oxidative stress and inflammatory response of the gastrointestinal tract	[100]
		Tannic acid, Ga^3+^	Etoposide	250 nm	Suppress tumor growth and prolong mice survival	[101]
	EGCG	EGCG, Cu2^+^	diethyldithiocarbamate	4.5 ± 0.8 nm	Inhibit multiple antibiotic-resistant Gram-positive bacterial strains	[102]
**Biologic drug**	Tannic acid	Tannic acid, tetrahedral framework nucleic acid	siRNA	15.20 ± 2.33 nm	Reduce inflammation and alleviate psoriasis-like symptoms in a mouse model	[103]
		Tannic acid	Superoxide dismutase and catalase	50 nm	Inhibit pyroptosis Reduce inflammation Decrease neutrophil infiltration into the liver	[104]
		Tannic acid, bovine serum albumin, chitosan, and gallic acid–mediated graphene quantum dot	TNF-α siRNA	230–350 nm	Reduce inflammatory bowel disease	[105]
		Tannic acid, glycidyl methacrylate-modified carboxymethyl chitosan	Antagomir-21	260 nm	Promote nucleus pulposus regeneration Delay intervertebral disc degeneration progression	[106]
		Tannic acid, branched-DNA	antisense DNA and DNAzyme	150 nm	Suppress tumor growth	[107]
		Tannic acid, Mn^2+^	DNAzyme	170 ±16 nm	Enhance antitumor effects and rate of mRNA silencing	[108]
**Multiple drugs**	Tannic acid	Tannic acid, Fe^3+^, Mn^2+^	Methylene blue and Ovalbumin	160 nm	Promote anti-tumor immune response	[109]
	Dopamine	Dopamine, Fe^3+^, oligo(ethylene glycol) methacrylate and acrylic acid	Doxorubicin and glucose oxidase	159 nm	Enhance inhibition of tumor growth	[110]

## Data Availability

No new data were created or analyzed in this study.

## Supplementary Materials

The following supporting information can be downloaded at: https://www.mdpi.com/article/10.3390/molecules31061051/s1, Table S1: Immunomodulatory effect of polyphenol.
