# Supplementary material for: Polyphenol-Based Nanomedicine: Versatile Platforms for Immune Modulation and Therapeutic Delivery
Source: Molecules. 2026 Mar 22;31(6):1051. doi: 10.3390/molecules31061051 (PMC13029208; doi:10.3390/molecules31061051)
Supplement: Supplementary file 1 [file molecules-31-01051-s001.zip › molecules-4138874-supplementary.pdf]

Table S1. Immunomodulatory effect of polyphenol

Immune cells	Polyphenol	Activities	Mechanism	Ref.
Dendritic cells	Curcumin	Prevent DCs from antigen presentation	Reduce the expression of co-stimulatory and adhesion molecules on DCs	[6]
	EGCG	Enhance DCs phagocytosis	Activate the toll-like receptor 4-dependent signaling in DCs	[4]
	Silibinin	Suppress the activation of DCs or pathways related to DCs	- Reduce the upregulation of costimulatory and MHC molecules<br>- Inhibit interleukin (IL)-12, IL-23, and tumor necrosis factor-alpha (TNF-α) production	[5]
Macrophages	Curcumin	Promote M2 macrophage polarization	- Increase IL-4, IL-10, and CD206<br>- Decrease IL-1β, TNF-α, C-C chemokine receptor type 7, and inducible nitric oxide synthase<br>- Inhibit the toll-like receptor 4/myeloid differentiation factor 88/ nuclear factor kappa B (NF-κB) signaling pathway	[9,10]
	Tannic acid	- Decrease M2-type macrophages<br>- Enhance the antitumor immune response	Regulate the reprogramming of tumor-associated macrophages from the M2 phenotype to an anti-tumoral, immune-activating phenotype	[15]
	Gallic acid	Promote M2 macrophage polarization	Enhance the expression of genes related to mitochondrial oxidative phosphorylation, metabolic regulation, and the PI3K–Akt signaling pathway.	[11]
	Resveratrol	- Promote M2 macrophage polarization<br>- Accelerate the healing of diabetic wounds<br>- Facilitate tissue repair and functional recovery	- Activate the PI3K-AKT signaling pathway<br>- Decrease TNF-α, IL-6, and IL-1β<br>- Inhibit apoptosis and ferroptosis	[12–14]
T cells	Curcumin	- Reduce the uncontrolled inflammation in acute lung injury/acute respiratory distress syndrome<br>- Delay multiple sclerosis development	- Promote the differentiation of CD4+ T cells into CD4+ CD25+ FOXP3+ regulatory T cells (Tregs)<br>- Stimulate Treg and T helper type 2 (Th2) cell polarization<br>- Activate the differentiation of Treg cells that might regulate IL-35	[17–19]
	Fisetin	Alleviate Psoriasis-like Skin Inflammation	- Suppress IL-17A secretion from activated CD4+ T cells<br>- Inhibit T-lymphocytes and F4/80+ macrophage infiltration into the skin	[23]
	Oleuropein	Ameliorate rheumatoid arthritis	Increase the frequency of CD4+CD25+FoxP3+ Tregs	[24]

Immune cells	Polyphenol	Activities	Mechanism	Ref.
	Procyanidin	- Reduce intracellular glutamine levels in T CD4+ cells<br>- Decrease the number of interferon-gamma (IFN-γ) cytokines<br>- Attenuate inflammatory responses	- Interact directly with the glutamine transporter to inhibit glutamine influx<br>- Control the cytokine network associated with Th17 cells<br>- Decrease IL-17, TNF-α, IL-1β, and IL-6 with the suppression of transcription factors expression	[20,21]
	Quercetin	Modulate many stages of osteoclastogenesis	Inhibit Th17 cell differentiation	[22]
	Resveratrol	- Suppress hepatocellular carcinoma progression<br>- Enhance anti-tumor activity and T cell activation<br>- Reverse T cell-mediated immune response	- Reduce CD8+CD122+ Tregs and M2-macrophages<br>- Antagonize with the aryl hydrocarbon receptor<br>- Inhibit Arginase-1 and C-X-C chemokine receptor type 2 expression	[25–27]
Neutrophils	Resveratrol	Attenuate neutrophil-mediated oxidative stress and inflammatory responses	Inhibit endothelin-mediated autocrine signaling	[31]
	Gallic acid	Alleviate neutrophil-driven inflammation	Modulate redox sensitive transcription factors, NF-κB and nuclear factor erythroid 2-related factor 2	[30]
Natural killer (NK) cells	Resveratrol	- Enhance NK cell cell-mediated antiviral and antitumor responses<br>- Suppress viral replication and improve immune defense	- Promote silent information regulator 1 and p53 signaling pathways<br>- Activate cytotoxic T lymphocytes and NK cells<br>- Regulate pro-inflammatory cytokines as IL-1β, IFN-γ and TNF-α	[33,34]
